# Absence of Embigin accelerates hearing loss and causes sub-viability, brain and heart defects in C57BL/6N mice due to interaction with *Cdh23*^*ahl*^

**DOI:** 10.1016/j.isci.2023.108056

**Published:** 2023-09-26

**Authors:** Sherylanne Newton, Carlos Aguilar, Rosie K. Bunton-Stasyshyn, Marisa Flook, Michelle Stewart, Walter Marcotti, Steve Brown, Michael R. Bowl

**Affiliations:** 1 Mammalian Genetics Unit, Medical Research Council Harwell Institute, Harwell Oxford, Oxfordshire OX11 0RD, UK; 2 UCL Ear Institute, University College London, 332 Gray’s Inn Road, London WC1X 8EE, UK; 3 The Mary Lyon Centre, Medical Research Council Harwell Institute, Oxford, Oxfordshire OX11 0RD, UK; 4 School of Biomedical Science, University of Sheffield, Sheffield S10 2TN, UK; 5 Sheffield Neuroscience Institute, University of Sheffield, Sheffield S10 2TN, UK

**Keywords:** Neurogenetics, Developmental genetics, Phenotyping, Model organism

## Abstract

Mouse studies continue to help elaborate upon the genetic landscape of mammalian disease and the underlying molecular mechanisms. Here, we have investigated an *Embigin*^*tm1b*^ allele maintained on a standard C57BL/6N background and on a co-isogenic C57BL/6N background in which the *Cdh23*^*ahl*^ allele has been “repaired.” The hypomorphic *Cdh23*^*ahl*^ allele is present in several commonly used inbred mouse strains, predisposing them to progressive hearing loss, starting in high-frequency regions. Absence of the neural cell adhesion molecule Embigin on the standard C57BL/6N background leads to accelerated hearing loss and causes sub-viability, brain and cardiac defects. Contrastingly, *Embigin*^*tm1b/tm1b*^ mice maintained on the co-isogenic “repaired” C57BL/6N background exhibit normal hearing and viability. Thus *Embigin* genetically interacts with *Cdh23.* Importantly, our study is the first to demonstrate an effect of the common *Cdh23*^*ahl*^ allele outside of the auditory system, which has important ramifications for genetic studies involving inbred strains carrying this allele.

## Highlights

• The Basigin-family member *Embigin* genetically interacts with *Cadherin23*
• *Embigin*-KO mice exhibit auditory, brain and heart defects when *Cdh23*^*ahl*^ is present
• Loss of *Embigin* causes subviability in C57BL/6N mice carrying the *Cdh23*^*ahl*^ allele
• The *Cdh23*^*ahl*^ allele, common to many inbred mouse strains, affects more than hearing

## Graphical abstract

**Figure:**
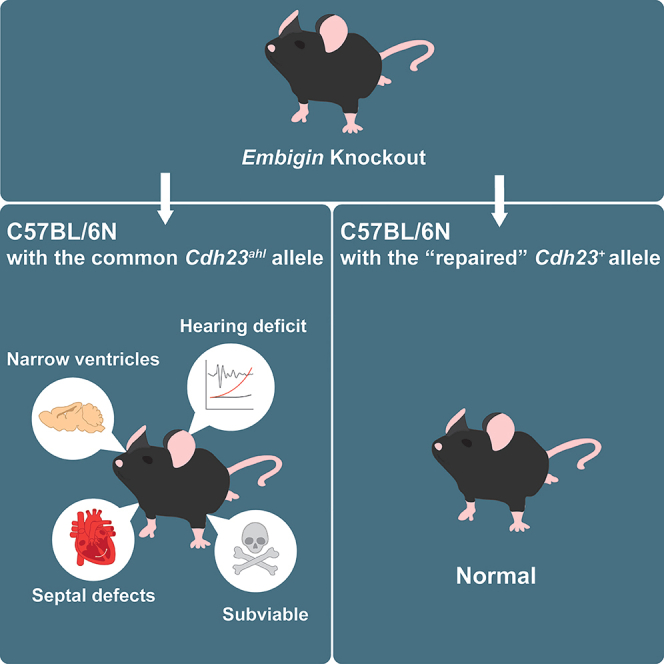

## Introduction

Hearing impairment is the most common sensory deficit in the human population, affecting some 430 million people worldwide. In developed countries, the occurrence of hearing loss in prelingual children is reported to be ∼1/500, and in these cases up to 80% may have a genetic basis.^1^ In addition, hearing loss can arise as a late-onset progressive condition reflecting cochlear aging combined with additional factors such as genetic predisposition.^2^ In humans, around 190 independent loci have been identified to be associated with non-syndromic hearing loss and causative genes have been identified for ∼70% of these. The similarities between human and mouse auditory structure and function, and the concordance between orthologous genes critical for hearing function, has seen the mouse become the predominant model organism for elaborating upon the genetic landscape of hearing and for interrogating the pathophysiology associated with gene mutations.^3^^,^^4^ In particular, highly genetically controlled inbred mouse strains have been instrumental in this endeavor, either through the identification and characterization of spontaneously arising audiovestibular mutants, or as a host into which targeted gene mutations can be introduced and phenotypes assessed and compared.^4^ Importantly, the strict genetic control afforded by inbred mouse strains allows for the identification of subtle additive phenotypic effects, such as those caused by genetic interaction.

The *Embigin* gene (*Emb*) encodes a highly glycosylated protein belonging to a small subgroup of immunoglobulin neural cell adhesion molecules alongside *Basigin* (*Bsg*, also known as CD147) and *Neuroplastin* (*Nptn*), the latter of which has recently been linked to early onset hearing loss in mice.^5^^,^^6^^,^^7^ Embigin was first described as a protein expressed at high levels during the development of several different tissue types.^8^ Thus, it is thought to play a role in the early stages of embryogenesis. Low levels of expression are also found in a number of adult rodent tissues; in particular, in the brain, the ovary, and the uterus during pregnancy.^9^ Data from the International Mouse Phenotyping Consortium (IMPC) identified the Embigin mutant (*Emb*^*tm1b(KOMP)Wtsi*^) as a candidate for high-frequency hearing loss, reporting elevated hearing thresholds in the two homozygous mice tested at 14-week of age as part of the adult phenotyping pipeline.^3^ Given the high-throughput nature of the IMPC program, additional investigation and characterization of the *Emb* mutant mice are required to confirm if *Embigin* is a gene required for hearing.

The IMPC program aims to generate and phenotype a knockout mouse mutant for every coding gene in the mouse genome. To enable the comparison of phenotyping data across centers, all mutants are generated on an inbred C57BL/6N genetic background. This strain, together with the closely related and commonly used C57BL/6J inbred strain, exhibits a form of accelerated age-related hearing loss (ARHL) characterized by high-frequency hearing loss beginning between 3-to-6 months of age. This then progresses to profound hearing impairment by 15-month of age. The cause of the accelerated hearing loss is due to a strain-specific single nucleotide variant within the *Cadherin 23* (*Cdh23*) gene, which encodes a component of the stereocilia tip link that is essential for mechanoelectrical transduction of sound.^10^ This hypomorphic allele, *Cdh23*^*c.753A*^ (*Cdh23*^*ahl*^), causes in-frame skipping of the seventh coding exon of *Cdh23* (ENSMUSE00001269485), and is present in >20 different inbred strains that display ARHL.^11^ Although the association between Cadherin 23 and the tip link is well established, mice homozygous for the *Cdh23*^*ahl*^ allele display a wide range of cochlear pathologies as they age such as degeneration of the spiral ligament and spiral ganglion neuron loss,^12^^,^^13^ alongside both biophysical and morphological changes to the inner and outer hair cells^14^^,^^15^ which occurs before degeneration.^16^^,^^17^ Thus far, the exact mechanism by which the *Cdh23*^*ahl*^ allele causes this accelerated degeneration is not yet known.^17^^,^^18^

Using C57BL/6N mice and a co-isogenic C57BL/6N strain, in which the *Cdh23*^*ahl*^ allele has been repaired using CRISPR/Cas9-mediated homology-directed repair (C57BL/6N.*Cdh23*^*c.753A>G*^), we recently demonstrated that the closely related gene *Neuroplastin* genetically interacts with the *Cdh23*^*ahl*^ allele potentiating the auditory deficit exhibited by *Neuroplastin* mutant mice.^7^ Here, we demonstrate that *Embigin* also genetically interacts with the *Cdh23*^*ahl*^ allele, causing high-frequency hearing deficits from as early as 1-month of age. However, unlike in the case of *Neuroplastin*, the cause of hearing impairment in the *Embigin* mutant is not due to a physical interaction with plasma membrane calcium ATPase (PMCA) proteins. In addition, we also find that co-expression of the *Cdh23*^*ahl*^ allele with the *Emb*^*tm1b(KOMP)Wtsi*^ allele results in embryonic brain and cardiac abnormalities, and an associated perinatal sub-viability. Thus, to our knowledge, this study is the first to demonstrate a role for the common strain-specific *Cdh23*^*ahl*^ allele in pathologies outside of the auditory system. This finding highlights the importance of considering the genetic background of mutant mice when characterizing phenotype expressivity, which is important given the widespread use of C57BL/6N and/6J mice in basic and translational research.

## Results

### Embigin is a cochlear expressed protein

*Embigin* (*Emb*) was previously reported as a candidate novel gene for hearing loss.^3^ However, due to the high-throughput nature of the IMPC, only two homozygous *Embigin* mutant mice underwent auditory brainstem response (ABR) testing. Additionally, a larger cohort of *Embigin* mutant mice (8 males and 8 females) were subject to acoustic startle and pre-pulse inhibition response testing. However, their responses were found to not be significantly different from those of age- and sex-matched control mice (https://www.mousephenotype.org/data/genes/MGI:95321).

**Figure 1:**
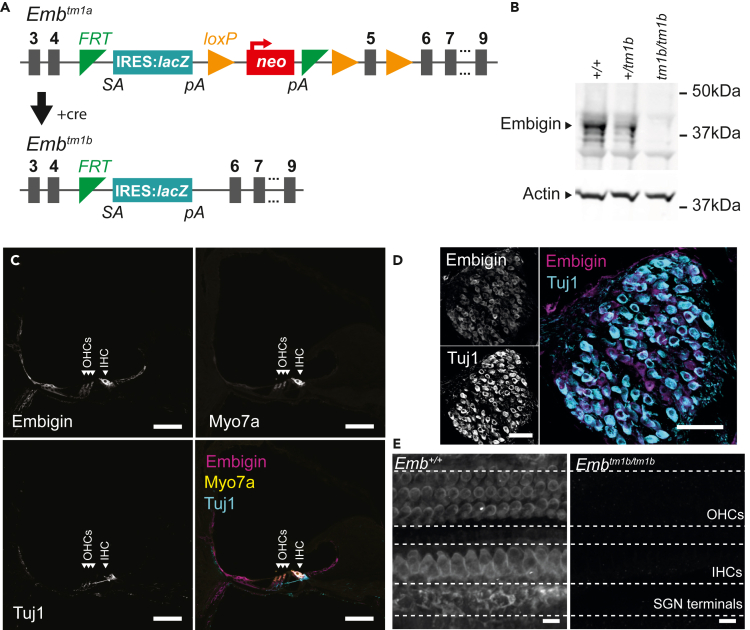
Embigin is expressed in the adult cochlea (A) Schematic of the conversion of the *Emb*^*tm1a*^ knockout-first allele to the *Emb*^*tm1b*^ knockout allele, through the Cre-mediated deletion of the *neo* selection cassette and *Embigin* exon 5. In the resultant *Emb*^*tm1b*^ allele there remains an IRES:lacZ trapping cassette. The trapping cassette includes the mouse En2 splice acceptor (SA) and the SV40 polyadenylation sequences (pA). The Internal Ribosome Entry Site (IRES) sequence sits between the En2 splice acceptor and the *lacZ* gene sequence. Thus, from a single transcript driven by the Embigin promoter, the presence of the IRES allows the independent translation of β-galactosidase. In addition, when the En2 splice acceptor is utilized, exon 4 of Embigin is not in-frame to the ATG of *lacZ*, and as such translation of a fusion protein is not possible. (B) Western blot utilizing *Emb*^*+/+*^, *Emb*^*+/tm1b*^, and *Emb*^*tm1b/tm1b*^ whole cochlear lysates. Lysates were treated with PNGase F to remove N-linked glycans prior to separation and blotting. Subsequently, blots were probed with the G7.43.1 Embigin monoclonal antibody. In the wild type lysate lane this produced a strong signal at approximately 37 kDa, corresponding to native Embigin. However, in the *Emb*^*+/tm1b*^ lysate lane the relative intensity of the ∼37 kDa band is reduced. In the *Emb*^*tm1b/tm1b*^ lysate lane there is no signal at ∼37 kDa, confirming *Emb*^*tm1b/tm1b*^ is a knockout model. See also Figure S1. (C and D) Cochlear cryosections from 4-week old wild-type mice, labeled with the G7.43.1 anti-Embigin antibody (magenta), the hair cell specific marker Myo7a (yellow), and the neural marker β-Tubulin III (Tuj1, cyan). (C) Embigin labeling is detected within both outer and inner hair cells, in the medial and lateral supporting cells, and in the spiral prominance. (D) Embigin labeling is also detected within the spiral ganglion cell bodies. Scale bar, 50 μm. (E) Whole-mount immuno-labeling of the organ of Corti harvested from 6-week-old mice, labeled with the G7.43.1 anti-Embigin antibody. In the *Emb*^*+/+*^ cochlea, Embigin labeling is detected in both inner- (IHCs) and outer hair cells (OHCs), with expression restricted to the cell bodies. Embigin labeling is also present at the spiral ganglion neuron (SGN) terminals. Using the same conditions, no labeling is detected in *Emb*^*tm1b/tm1b*^ cochleae. Scale bar, 20 μm.

To further study the biological requirement of Embigin, mice carrying the *Embigin* “knockout” *Emb*^*tm1b(KOMP)Wtsi*^ allele, hereafter called *Emb*^*tm1b*^ were established. This allele contains a cassette inserted into intron 4, comprising an engrailed intron splice acceptor and *LacZ* reporter, and a deletion of exon 5 (Figure 1A). These changes are designed to generate an *Emb*^*tm1b*^ knockout allele. The splice acceptor should subsume splicing from *Embigin* exon 4, and the deletion of exon 5 would create a frameshift in the unlikely event of exon 4 splicing directly to exon 6. To ascertain if the *Emb*^*tm1b*^ allele is a knockout, western blotting was undertaken on cochlear lysates using two commercially available antibodies: the G7.43.1 anti-Embigin monoclonal antibody, and a polyclonal antibody raised against the C-terminus of Embigin. Blots using the polyclonal antibody produced a single 30 kDa band in wild-type brain lysates which does not correspond to any known Embigin isoform, whereas the monoclonal antibody produced dispersed bands around 70 kDa which was absent in homozygous lysates (Figure S1). Embigin is a highly glycosylated protein, as such total protein lysates were treated with PNGase F to remove *N*-linked oligosaccharides allowing the detection of proteins in their unmodified, native state. Deglycosylated Embigin has a molecular weight of approximately 37 kDa, rather than up to 70 kDa in its glycosylated form. In wild type cochlear tissues, a distinct band was detected at approximately 37 kDa. The relative intensity of this band was reduced in *Emb*^*+/tm1b*^-derived tissue lysates and absent in *Emb*^*tm1b/tm1b*^-derived tissue lysates (Figure 1B). These data demonstrate that Embigin protein is present in the murine cochlea, and confirms that the *Emb*^*tm1b/tm1b*^ mouse is a knockout model.

To study the expression pattern of Embigin, immunohistochemical labeling was performed on cochlear cryosections prepared from 4-week old wild-type mice. Embigin signal is detected within epithelial cells lining the cochlear duct, including the inner and outer sulcus. In addition, signal is present within inner and outer hair cells, as well as supporting cells, including Hensen, Boettcher, and Claudius cells. However, no signal was evident within the stria vascularis (Figure 1C). Immunolabeling was also observed within spiral ganglion cell bodies (Figure 1D). We also utilized the *lacZ* reporter cassette present in heterozygous *Emb*^*+/tm1b*^ mice, transcription of which is under the control of the endogenous *Embigin* promoter, to detect β-Gal-positive cells in the cochlea. The pattern of β-Gal expression was concordant with the fluorescence labeling obtained using the anti-Embigin antibody (Figure S2). To further probe expression in the region around the inner and outer hair cells, the G7.43.1 anti-Embigin antibody was employed for immunohistochemical labeling of cochlear whole-mount tissue harvested from 6-week-old mice. In wild-type tissue, a positive Embigin signal was observed in the membrane of both outer- and inner hair cells and the spiral ganglion afferent terminals (Figure 1E). There was no evidence that Embigin is present within the stereocilia of inner- or outer hair cells.

### *Embigin* is required for hearing and genetically interacts with *Cadherin 23*

To determine if Embigin has a role in mammalian hearing two studies were undertaken. First, to validate the preliminary findings of the IMPC the *Embigin* mutant (*Emb*^*tm1b*^) mice were maintained on a C57BL/6N background, and subjected to auditory threshold testing using ABR measurements. Second, to determine if *Embigin* interacts with the strain-specific *Cdh23*^*ahl*^ allele, we crossed the *Emb*^*tm1b*^ allele onto a C57BL/6N-repaired background, such that all mice within the colony were homozygous for the “repaired” *Cdh23* allele (C57BL/6N.*Cdh23*^*c.753A>G*^).^16^ To generate the experimental cohorts, heterozygous *Emb*^*+/tm1b*^ mice were intercrossed and the resultant offspring were subject to ABR phenotyping.

**Figure 2:**
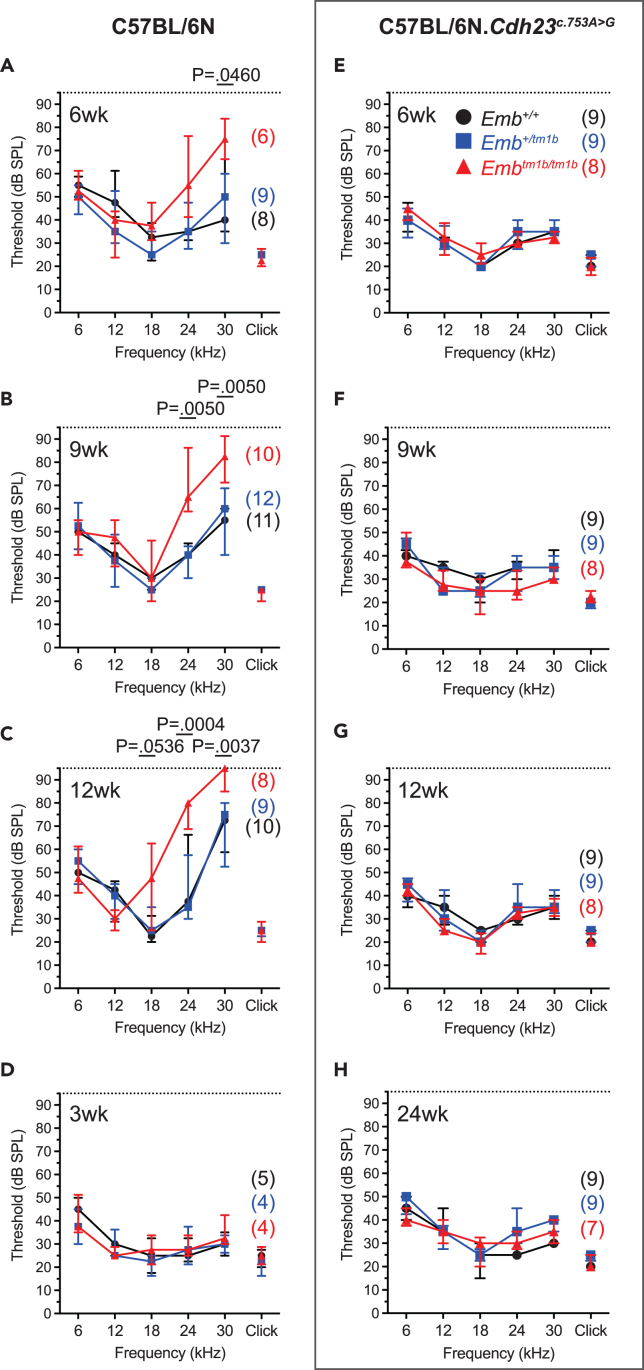
*Emb*^*tm1b/tm1b*^ mice exhibit an early onset progressive high-frequency hearing deficit that is contingent on the presence of the *Cdh23*^*ahl*^ allele (A–D) ABR threshold measures recorded from (A) 6-week, (B) 9-week, (C) 12-week, and (D) 3-week-old mice maintained on a C57BL/6N genetic background identify an early onset progressive elevation of the high-frequency ABR thresholds of *Emb*^*tm1b/tm1b*^ mice (red triangles) compared to *Emb*^*+/tm1b*^ (blue squares) and *Emb*^*+/+*^ (black circles) littermate mice. (E–H) ABR threshold measures recorded from 6-week (E), 9-week (F), 12-week (G), and 24-week (H) old mice maintained on a C57BL/6N.*Cdh23*^*c.753A>G*^ genetic background. ABR thresholds of *Emb*^*tm1b/tm1b*^ mice (red triangle) are comparable to those of their littermate controls, *Emb*^*+/tm1b*^ (blue squares), and *Emb*^*+/+*^ (black circles), at all ages tested. Data shown are median ± I.Q.R. Statistical analysis performed using a two-way ANOVA comparing against wild type with Holm-Šídák’s test for multiple comparisons. Number of mice tested for each time point shown in brackets.

For mice maintained on the C57BL/6N background, the 30 kHz auditory thresholds of homozygous *Emb*^*tm1b/tm1b*^ mice (73 ± 18 dB SPL, n = 6) are elevated compared to their wild type littermate control *Emb*^*+/+*^ mice at 6-week of age (47 ± 18 dB SPL, n = 8, p = 0.0460, two-way ANOVA with Holm-Šídák’s post-test for multiple comparisons) (Figure 2A). This high-frequency hearing loss is progressive, with ABR thresholds of *Emb*^*tm1b/tm1b*^ mice maintained on the C57BL/6N background being further elevated by 9-week of age at 24 kHz (p = 0.0050) and 30 kHz (p = 0.0050) (Figure 2B). Furthermore, by 12-week of age the ABR thresholds are elevated further at 24 kHz (p = 0.0004) and 30 kHz (p = 0.0037), and the average hearing threshold for the 18 kHz stimulus (46 ± 20 dB SPL, n = 8) is also elevated compared to that of *Emb*^*+/+*^ littermate controls (25 ± 7 dB SPL, n = 10). However, the degree of hearing loss at 18 kHz is highly variable across the *Emb*^*tm1b/tm1b*^ group, and is not statistically significant to their wild type littermate controls (p = 0.0536) (Figure 2C). Comparisons between ABR thresholds of male and female homozygous *Emb*^*tm1b/tm1b*^ ABR thresholds at 6- and at 9-week of age showed no difference between sexes (Figure S3). These data are consistent with those found by the IMPC program, which reported elevated thresholds at 18-, 24-, and 30 kHz at 14-week of age,^3^ and confirm that Embigin is important for mammalian hearing. Note the less severe, progressive high-frequency hearing loss exhibited by the wild type and heterozygous littermate mice at 12 weeks compared with the same mice at 6 weeks of age, which is expected in wild type C57BL/6N mice and results from the presence of the *Cdh23*^*ahl*^ allele in this background. To determine if the hearing loss exhibited by *Emb*^*tm1b/tm1b*^ mice on the C57BL/6N background is congenital, a second cohort of mice was generated to test the ABR thresholds of mice at 3-week of age were recorded, which is approximately 1-week after mice would normally begin to hear. At this age both *Emb*^*+/tm1b*^ and *Emb*^*tm1b/tm1b*^ mice exhibit ABR thresholds that are indistinguishable from their *Emb*^*+/+*^ control littermates (Figure 2D), suggesting auditory development and maturation occurs as normal in these mice.

For mice maintained on the “repaired” C57BL/6N*.Cdh23*^*c.753A>G*^ background, ABR phenotyping at 6-, 9-, and 12-week of age shows that ABR thresholds of *Emb*^*tm1b/tm1b*^ mice are indistinguishable from their age-matched wild type and heterozygous littermate controls at all measured frequencies (Figures 2E–2G). To determine if a high-frequency hearing loss phenotype develops later in *Emb*^*tm1b/tm1b*^ mice maintained on the C57BL/6N*.Cdh23*^*c.753A>G*^ background, mice were aged to 24-week of age and subject to hearing threshold assessment. At this age *Emb*^*tm1b/tm1b*^ mice still exhibit ABR thresholds that are indistinguishable from their age-matched littermate controls at all measured frequencies (Figure 2H). Note that the high-frequency ABR thresholds are maintained across all genotypes between 6- and 12-week, which is due to the absence of the *Cdh23*^*ahl*^ allele in the “repaired” C57BL/6N background. These data indicate that *Emb*^*tm1b/tm1b*^ mice maintained on a C57BL/6N background initially have normal ABR thresholds, but then exhibit an early onset progressive high-frequency hearing loss. Interestingly, this phenotype is contingent on the presence of the *Cdh23*^*ahl*^ allele, as *Emb*^*tm1b/tm1b*^ mice maintained on the “repaired” C57BL/6N*.Cdh23*^*c.753A>G*^ background do not exhibit any elevation in their ABR thresholds, at least up to 24-week of age.

### *Emb*^*tm1b*^ mutants exhibit no gross cochlear dysmorphology

**Figure 3:**
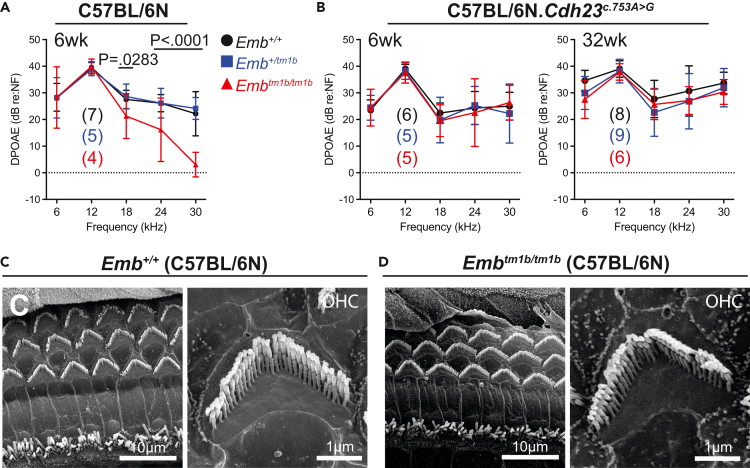
C57BL/6N *Emb*^*tm1b/tm1b*^ mice have reduced outer hair cell function (A) DPOAE responses recorded from mice maintained on a C57BL/6N background at 6-week of age show reduced responses in *Emb*^*tm1b/tm1b*^ mice compared to *Emb*^*+/tm1b*^ and *Emb*^*+/+*^ controls at frequencies >18 kHz. (B) DPOAE responses recorded from mice maintained on the “repaired” C57BL/6N background at 6- and 32-week of age show no significant difference between *Emb*^*tm1b/tm1b*^ (red triangles)*, Emb*^*+/tm1b*^ (blue squares) and *Emb*^*+/+*^ (black circles) mice. (Re:NF = value relative to noise floor). DPOAE data are mean ± S.D. Statistical analysis performed using a two-way ANOVA comparing against wild type with Holm-Šídák’s test for multiple comparisons. Number of mice tested shown in brackets. (C and D) SEM images taken of the mid-basal cochlear sensory epithelia of *Emb*^*+/+*^ and *Emb*^*tm1b/tm1b*^ mice, maintained on a C57BL/6N background, harvested at 6-week of age. This region of the cochlea, 2/3rds of the distance from apex to base, is tonotopically tuned to approximately 30 kHz. *Emb*^*tm1b/tm1b*^ cochleae show no notable loss of inner- or outer hair cell bundles. High-magnification SEM images show no observable difference in the morphology of outer hair cell stereocilia bundles in *Emb*^*+/+*^ and *Emb*^*tm1b/tm1b*^ cochleae.

When maintained on a C57BL/6N background an auditory deficit is evident in *Emb*^*tm1b/tm1b*^ mice consisting of elevated ABR thresholds for high-frequency (30 kHz) stimuli, at 6-week of age. This phenotype might suggest reduced function, or loss, of cochlear hair cells. Firstly, outer hair cell (OHC) function was assessed *in vivo* using distortion product otoacoustic emissions (DPOAEs). At 6-week of age, *Emb*^*tm1b/tm1b*^ mice have reduced DPOAE responses compared to their *Emb*^*+/+*^ controls at 18 kHz (p = 0.0283, two-way ANOVA, with Holm-Šídák’s post-test for multiple comparisons), 24 kHz and 30 kHz (p < 0.0001) (Figure 3A). These data suggest a deficit in the function of outer hair cells located toward the base of the cochlea in *Emb*^*tm1b/tm1b*^ mice maintained on a C57BL/6N background, which is tonotopically concordant with the increased high-frequency ABR thresholds recorded at this age. In contrast, no differences in DPOAE responses were evident in *Emb*^*tm1b/tm1b*^ mice maintained on the “repaired” C57BL/6N.*Cdh23*^*c.753A>G*^ background compared to their littermate controls when measured at 6- or 32-week of age (Figure 3B).

On the C57BL/6N background scanning electron microscopy (SEM) was utilized to assess the architecture of the cochlear sensory epithelium to determine if the reduced DPOAE responses recorded in the *Emb*^*tm1b/tm1b*^ mice are due to a cellular loss or dysmorphology. At 6-week of age, *Emb*^*tm1b/tm1b*^ mice have no overt inner hair cell (IHC) or OHC stereocilia bundle defects, nor is there any loss of hair cells (as determined by the presence of stereocilia bundles), with bundle organization and hair cell numbers comparable to their wild type littermates (Figures 3C and 3D).

**Figure 4:**
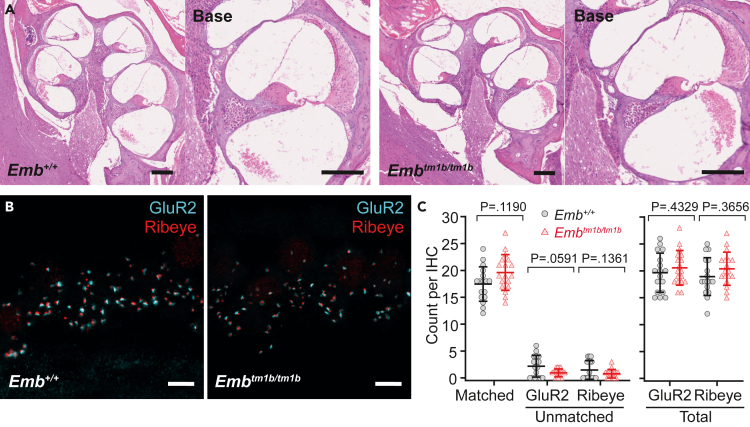
C57BL/6N *Emb*^*tm1b/tm1b*^ mice have no gross cochlear morphological defects (A) Histological sections stained with hematoxylin and eosin prepared from mice at 24-week of age. *Emb*^*+/+*^ (left) and *Emb*^*tm1b/tm1b*^ (right) cochleae have comparable morphology at all regions, including the base, with no evidence of cellular degeneration. A higher magnification view of the basal turn for each genotype is also shown. Scale bar, 100 μm. (B) Basal cochlear whole-mounts from *Emb*^*+/+*^ and *Emb*^*tm1b/tm1b*^ mice at 6-week of age, labeled with the IHC pre-synaptic ribbon marker Ribeye (red) and the post-synaptic density marker GluR2 (cyan). Scale bar, 5 μm. (C) Counts of Ribeye and GluR2 puncta were made from 17 (*Emb*^*+/+*^) and 18 (*Emb*^*tm1b/tm1b*^) individual basal inner hair cells across 3 mice per genotype. To assist with the identification of individual hair cells, cochlear whole-mounts were labeled with a Myo7a antibody, which has not been included in these images. Ribeye and GluR2 were considered matched when directly juxtaposed to one another. No significant difference was found in the total numbers of Ribeye or GluR2 puncta, or matched Ribeye/GluR2 puncta. Data are mean ± S.D with individual data points shown. Data compared against wild type controls using unpaired t-tests with a Holm-Šídák’s correction for multiple comparisons.

Furthermore, histological sections prepared from 24-week old *Emb*^*+/+*^ and *Emb*^*tm1b/tm1b*^ mice maintained on a C57BL/6N background showed normal cochlear morphology in the *Emb*^*tm1b/tm1b*^ cochleae, with no evidence for loss or degeneration of: hair cells; spiral ganglion neurons; stria vascularis or supporting cells (Figure 4A). Given that we have recently reported significant IHC synaptic deficits in the Embigin-related Neuroplastin null mice,^7^ synaptic coupling between IHCs and afferent terminals of spiral ganglion neurons in *Emb*^*tm1b*^ mice maintained on a C57BL/6N background was assessed by whole-mount immuno-labeling.^5^^,^^7^ Co-labeling with anti-Ribeye (pre-synaptic ribbon marker) and anti-GluR2 (post-synaptic density marker) revealed no evidence of a synaptic deficit with similar numbers of matched, unmatched, and total number of puncta per IHC observed in *Emb*^*tm1b/tm1b*^ mice as compared to wild type littermates at 6-week of age (Figures 4B and 4C). Importantly, this time point was chosen for the analysis of the stereocilia bundle and synapse morphology as it precedes any effects attributable to the hypomorphic *Cdh23*^*ahl*^ allele.^16^^,^^17^ ABR Click wave I waveforms, which represent the summed sound stimuli-evoked electrical activity in the auditory nerve, were also assessed at 6- and 12-week of age. However, with the exception of a slight delay in latency at low intensities, the amplitudes and latencies exhibited by *Emb*^*tm1b/tm1b*^ mice were broadly comparable to their littermate controls (Figure S4). Together, these data suggest that a reduced OHC output is the primary cause of hearing loss in these *Emb*^*tm1b/tm1b*^ mutants.

### Embigin does not interact with PMCAs in the cochlea

**Figure 5:**
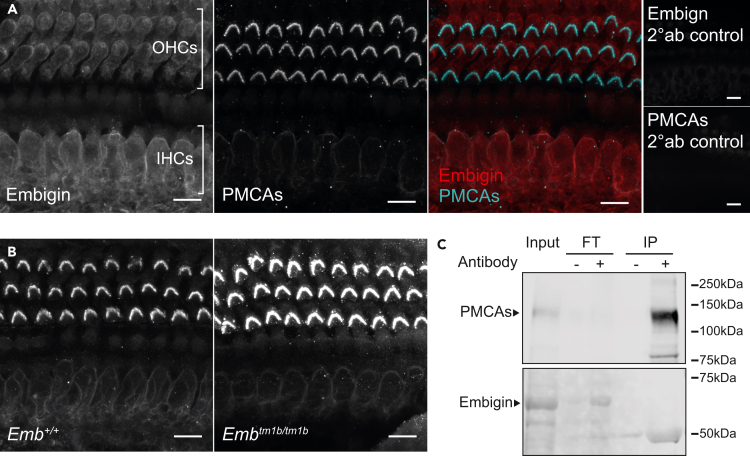
Embigin does not directly interact with PMCAs in the cochlea (A) Whole-mount immunohistochemistry of cochleae from 1-month old wild type mice (C57BL/6N background, *Cdh23*^*ahl/ahl*^) using anti-Embigin and anti-pan-PMCA antibodies. Signal for anti-Embigin is present in the cell membrane and diffusely within the cell body of IHCs and OHCs, whereas anti-PMCA signal is primarily restricted to the OHC stereocilia with some faint expression in IHC cell membranes. Scale bar, 10 μm. (B) Whole-mount immunohistochemistry of cochleae from 1-month old *Emb*^*+/+*^ and *Emb*^*tm1b/tm1b*^ (C57BL/6N background) mice using anti-pan-PMCA antibody. There is no observable reduction of anti-PMCA signal in mutant *Emb*^*tm1b/tm1b*^ cochleae compared to wild type cochleae. Scale bar, 10 μm. (C) Co-immunoprecipitation of whole cochlear lysates, obtained from wild type C57BL/6N mice, using the pan-PMCA (5F10) antibody to isolate proteins physically interacting with PMCA1-4. PMCAs (∼120 kDa) and Embigin (∼70 kDa) were detected in the total protein lysate (Input) lane. Compared to Input, PMCAs were enriched in the immunoprecipitate (IP) lane. Embigin protein was detected in the flow through (FT), but is not detected in the IP lane. Positive (+) denotes lanes which had the pan-PMCA antibody included, whereas negative (−) denotes lanes where the pan-PMCA antibody was excluded.

Two of the three members of the Basigin immunoglobulin family, namely Basigin and Neuroplastin, have recently been identified as obligatory subunits of PMCA proteins.^19^^,^^20^^,^^21^ In particular, Neuroplastin has been shown to be critical for the transport of PMCA1 and PMCA2 to the cell membrane of cochlear hair cells, with this function being dependent on the transmembrane domain of Neuroplastin.^7^^,^^22^ Interestingly, when investigated in brain tissues, Schmidt et al.^21^ could find no evidence of an interaction between PMCAs and Embigin, the third family member. This is in spite of Embigin containing a transmembrane domain that shows high levels of sequence conservation with the transmembrane domain of Neuroplastin.^23^ Regardless of this, the high-frequency hearing loss exhibited by *Emb*^*tm1b/tm1b*^ mice bears similarity to the recently described auditory deficit of heterozygous *Nptn* knockout mice, in that their phenotype is only evident when maintained on a C57BL/6N background *i.e*., in the presence of the *Cdh23*^*ahl*^ allele.^7^ The genetic interaction between *Nptn* and *Cdh23* is presumed to be a result of the direct physical interaction of Neuroplastin with PMCA1 and with PMCA2. To further explore a possible interaction between Embigin and PMCAs, we first undertook immuno-labeling experiments of cochlear whole-mounts prepared from wild-type mice. These showed very little overlap between the signals obtained for Embigin and PMCAs (Figure 5A). In addition, unlike reported for Neuroplastin-knockout tissues, there is no apparent reduction in cell surface localization of PMCAs in *Emb*^*tm1b/tm1b*^ cochlear hair cells (Figure 5B). To further investigate whether Embigin physically interacts with PMCAs, PMCA complexes were isolated from whole cochlear lysates obtained from wild type C57BL/6N mice by co-immunoprecipitation using the pan-PMCA (5F10) antibody. While PMCAs were enriched in the immunoprecipitate (IP), there is no evidence of a band corresponding to Embigin, suggesting there is not a direct interaction between Embigin and PMCAs in cochlear tissues (Figure 5C). This is consistent with the findings of Schmidt et al.^21^ who reported that PMCAs interacted with Neuroplastin and Basigin, but not Embigin, in neural tissues.

### Presence of the *Cdh23*^*ahl*^ allele does not affect *Embigin* expression

As *Embigin*^*tm1b/tm1b*^ mice only exhibit accelerated progressive hearing loss in the presence of the *Cdh23*^*ahl*^ allele, we assessed whether the *Cdh23*^*ahl*^ allele has an effect on the expression of *Embigin*. Utilizing quantitative RT-PCR, we generated four independent standard curves for *Emb*, and a housekeeping gene *Hprt*, using TaqMan assays. The coefficients of determination (R2) varied between from 0.997 to 1, and the amplification efficiencies ranged between 92.465% and 112.929%. We compared the expression of *Emb* in cochlear total RNA samples prepared from 7 “repaired” wild type (C57BL/6N.*Cdh23*^*c.753A>G*^) mice (3 males and 4 females) with 8 “standard” wild type (C57BL/6N) mice (4 males and 4 females) at 4-week of age. We observed no difference in *Emb* transcript level between repaired (ΔCt = −1.128 ± 0.234) and standard C57BL/6N mice (ΔCt = −1.134 ± 0.268) (p = 0.965, CI = [-0.279, 0.290]) (Figure S5). Thus, the *Cdh23*^*ahl*^ allele has no direct influence on the levels of *Embigin* transcripts in the cochlea.

### Presence of the *Cdh23*^*ahl*^ allele affects viability in *Emb*^*tm1b/tm1b*^ mice

**Figure 6:**
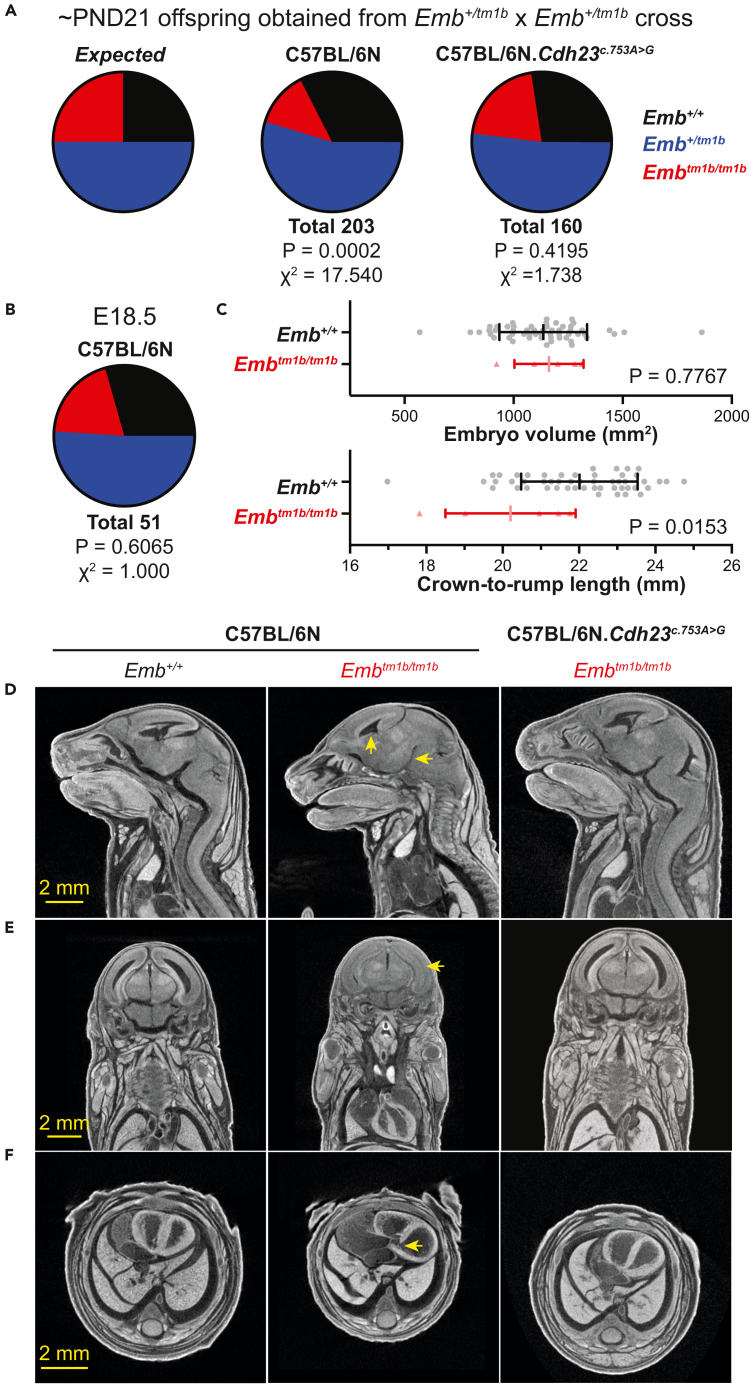
*Embigin* genetically interacts with *Cadherin 23* causing sub-viability and embryonic abnormalities (A) Mendelian genetics predicts that when intercrossing heterozygotes the offspring produced should comprise 25% wild type, 50% heterozygotes, and 25% homozygotes. On the C57BL/6N background (*Cdh23*^*ahl/ahl*^), heterozygote intercrosses produced 203 offspring that were genotyped at or before PND21. Of these 32.5% were *Emb*^*+/+*^, 54.7% were *Emb*^*+/tm1b*^, and 12.8% were *Emb*^*tm1b/tm1b*^. At these percentages, *Emb*^*tm1b/tm1b*^ mice are considered significantly sub-viable (p = 0.0002, Chi-Squared test, χ^2^ = 17.540). On the “repaired” C57BL/6N.*Cdh23*^*c.753A>G*^ background, heterozygote intercrosses produced 160 offspring that were genotyped at or before PND21. Of these 27.5% were *Emb*^*+/+*^, 51.9% were *Emb*^*+/tm1b*^, and 20.6% were *Emb*^*tm1b/tm1b*^. At these percentages, *Emb*^*tm1b/tm1b*^ mice are not significantly different from the expected number of 25% (p = 0.4195, Chi-Squared test, χ^2^ = 1.738). (B) On the C57BL/6N background, heterozygote intercrosses produced 51 E18.5 embryos, which were genotyped as: 29.4% *Emb*^*+/+*^; 51.0% *Emb*^*+/tm1b*^; and, 19.6% *Emb*^*tm1b/tm1b*^. These numbers are not significantly different from the expected Mendelian ratio (p = 0.6065, Chi-Squared test, χ^2^ = 1.000). (C) On the C57BL/6N background, embryo volume and crown-to-rump length measurements were ascertained from microCT scans of whole embryos harvested at E18.5. MicroCT measurements from 55 (30 female, 25 male) wild type C57BL/6N E18.5 embryos were collected and compared against scans from five *Emb*^*tm1b/tm1b*^ (three female, two male) embryos. On average, the embryo volume of *Emb*^*tm1b/tm1b*^ embryos was not significantly different when compared with wild type embryos (p = 0.7767, two-tailed unpaired t-test, t = 0.2849, df = 58). However, the crown-to-rump length of *Emb*^*tm1b/tm1b*^ embryos was significantly different when compared with wild type embryos (p = 0.0153, two-tailed unpaired t-test, t = 2.505, df = 54). (D–F) Whole body microCT scan sections from *Emb*^*+/+*^ and *Emb*^*tm1b/tm1b*^ E18.5 embryos. On the C57BL/6N background, 4 of the 5 *Emb*^*tm1b/tm1b*^ embryos displayed notable narrowing across several ventricular spaces of the brain (D and E). In addition, all 5 exhibited interventricular cardiac septum defects (F). On the “repaired” C57BL/6N.*Cdh23*^*c.753A>G*^ background, seven of the eight scanned *Emb*^*tm1b/tm1b*^ embryos showed normal morphology with no evidence of narrowed brain ventricles (D) nor cardiac septal defects (F). Regions indicated by yellow arrows. Scale bars, 2 mm.

While generating the experimental cohorts for ABR testing, it became apparent that by the age of weaning (∼postnatal day 21, PND21) there were fewer than expected *Emb*^*tm1b/tm1b*^ mice obtained on the C57BL/6N background compared to those maintained on the “repaired” C57BL/6N.*Cdh23*^*c.753A>G*^ background. On the C57BL/6N background, only 12.8% of genotyped mice (26 of 203) were found to be *Emb*^*tm1b/tm1b*^, which is significantly reduced compared with the 25% expected from a heterozygous cross (p = 0.0002, Chi-Squared test, χ^2^ = 17.540) (Figure 6A). These data suggest that *Emb*^*tm1b/tm1b*^ mice are sub-viable on the standard C57BL/6N background. In contrast, on the “repaired” C57BL/6N.*Cdh23*^*c.753A>G*^ background 20.6% of genotyped mice (33 of 160) were found to be *Emb*^*tm1b/tm1b*^, which is not significantly different from the expected 25% (p = 0.4195, Chi-Squared test, χ^2^ = 1.738) (Figure 6A).

To further investigate the decreased viability observed in *Emb*^*tm1b/tm1b*^ mice maintained on the C57BL/6N background, embryos from heterozygote crosses were harvested at embryonic day (E)18.5, when the day of birth (PND0) occurs between E19.5 and E20.5. Of the 51 embryos collected, 19.6% (10 of 51) were genotyped as homozygous (*Emb*^*tm1b/tm1b*^), which is not significantly different from the expected 25% (p = 0.6065, Chi-Squared test, χ^2^ = 1.000) (Figure 6B). Thus *Emb*^*tm1b/tm1b*^ mice maintained on the C57BL/6N background are still present at the expected Mendelian ratio at this late gestational time point. Furthermore, the percentage of *Emb*^*tm1b*^ mice lost between PND0 and PND14 in the *Emb*^*tm1b*^ colony was broadly comparable to the percentage lost in the C57BL/6N breeding colony maintained within the animal facility over a similar time period (*Emb*^*tm1b*^: 57/277, 20.5%; vs*.* C57BL/6N: 4821/26728, 18.0%). Thus *Emb*^*tm1b/tm1b*^ mice are likely lost in the perinatal period, where pup mortality is largely underestimated.^24^

To determine why *Emb*^*tm1b/tm1b*^ mice maintained on the C57BL/6N background are failing to survive, whole body microCT scans were performed on five E18.5 *Emb*^*tm1b/tm1b*^ embryos (three female, two male). For comparison, data were obtained from whole body microCT scans of 55 wild type C57BL/6N E18.5 embryos (30 female, 25 male) collected as part of the IMPC program using the same protocol, equipment, and operator.^25^^,^^26^ At E18.5, there is no significant difference between female and male wild type embryos when measuring volume (p = 0.1534, two-tailed unpaired t-test, t = 1.448, df = 53) or crown-to-rump length (CRL) (p = 0.3994, t = 0.8500, df = 49). As such, both sexes were pooled when comparing measurements taken from *Emb*^*tm1b/tm1b*^ embryos. On average, there is no significant difference in the volume of homozygous *Emb*^*tm1b/tm1b*^ embryos compared to the volume of wild type embryos (p = 0.7767, t = 0.2849, df = 58) (Figure 6C). However, on average, *Emb*^*tm1b/tm1b*^ embryos did exhibit a shorter CRL measurement compared to wild type embryos (p = 0.0153, t = 2.505, df = 54) (Figure 6C). The length of the *Emb*^*tm1b/tm1b*^ embryos did not correlate with biological sex, with the two shortest measurements of 19.02 mm and 17.82 mm taken from a female and male embryo, respectively.

Of the *Emb*^*tm1b/tm1b*^ embryos, four of the five displayed narrowing of one or more regions of the brain ventricles (Figures 6D and 6E). Additionally, all five embryos showed cardiac interventricular septum defects with two embryos each having an unambiguous opening between the ventricles (Figure 6F). In contrast, interrogation of whole body microCT scans performed on eight *Emb*^*tm1b/tm1b*^ E18.5 embryos maintained on the “repaired” C57BL/6N.*Cdh23*^*c.753A>G*^ background found that only one embryo displayed narrowed brain ventricles and severe cardiac hypertrophy (no evidence of septal defects). The remaining seven embryos had normal morphology with no evidence of cardiac defects nor narrowing of brain ventricles (Figures 6D–6F).

Together, these data suggest that *Emb*^*tm1b/tm1b*^ embryos are likely dying in the very late gestational period, or shortly after birth, due to developmental defects affecting the brain and heart. Interestingly, the sub-viability is associated with the presence of the *Cdh23*^*ahl*^ allele with the incidence of embryonic anomalies greatly increased on the C57BL/6N background compared to when maintained on the “repaired” C57BL/6N.*Cdh23*^*c.753A>G*^ background.

## Discussion

Here, we have shown that a second member of the Basigin-family of neural cell adhesion molecules, Embigin, is a cochlear expressed protein. It is present in a number of cells in the organ of Corti, including several types of supporting cells, and both the inner and outer hair cells (Figure 1). Furthermore, we demonstrate that the absence of Embigin in *Emb*^*tm1b/tm1b*^ mutant mice causes a progressive high-frequency hearing loss that manifests around adolescence (between 3-to-6 weeks of age). Intriguingly, this hearing deficit is dependent on the presence of the strain-specific *Cdh23*^*ahl*^ allele, which is present in several mouse strains, including the commonly utilized C57BL/6J and C57BL/6N strains. Indeed, C57BL/6 inbred mice have been extensively studied as a model of ARHL due to the presence of the *Cdh23*^*ahl*^ allele in these mice, which causes a progressive hearing loss phenotype beginning at high-frequencies from 3-to-6 months of age. Thus, our study identifies that the *Cdh23*^*ahl*^-induced high-frequency hearing loss phenotype is potentiated by the absence of Embigin, with the onset and progression of the hearing loss being evident at a much younger age in these mice.

### Embigin is a novel deafness gene in C57BL/6N mice

Our investigation of *Emb*^*tm1b*^ mutant mice on a C57BL/6N background shows that Embigin is required for maintaining auditory function (Figure 2), and validates data obtained from the high-throughput broad-based phenotyping undertaken by the IMPC program that reported *Embigin* as a candidate gene for high-frequency hearing loss.^3^ Auditory assessment of young *Emb*^*tm1b/tm1b*^ mice suggests that while auditory maturation is unaffected, hearing threshold increases are already evident from 6 weeks of age, with a concomitant reduction in DPOAE response output at the affected frequencies. While a reduction in otoacoustic emissions is indicative of OHC dysfunction, there was no gross dysmorphology of the OHC stereocilia bundles in *Emb*^*tm1b/tm1b*^ mice (Figure 3). Furthermore, localization of Embigin to inner- or OHC stereocilia was not suggested by immuno-labeling, with fluorescent signals restricted to the cell bodies of both hair cell types (Figure 1D). Thus, Embigin appears not to be required for stereocilia formation or maintenance, nor is Embigin likely to directly interact with the mechanoelectrical transduction machinery that is present at the tips of the shorter row stereocilia. While synaptic disruption is a key feature of loss of the related family member Neuroplastin,^5^^,^^7^ in the absence of Embigin there is no indication of synaptic mismatching (Figures 4B and 4C), nor any loss of neuronal cells as the animals age (Figure 4A). Thus, at this time the mechanism by which hearing loss is accelerated in the *Emb*^*tm1b*^ mutant mice is not clear.

In addition to hair cells, Embigin expression was also observed in cells comprising the inner- and outer-sulcus regions, with strong expression noted in the Hensen, Boettcher, Claudius cells, and cells of the spiral prominence. The presence of Embigin in these regions could point toward a role in cochlear fluid homeostasis, for which these cells have known importance.^27^ Indeed, Embigin is also reported to act as an obligatory subunit of proton-dependent L-lactate monocarboxylate transporters (MCTs), MCT1 and MCT2, in select tissue types.^28^^,^^29^^,^^30^ In addition to their role in cellular metabolism, these transporters are also involved in the maintenance of pH homoeostasis.^31^

### *Embigin* genetically interacts with the *Cdh23*^*ahl*^ allele in C57BL/6 mice

As mentioned, Embigin together with Neuroplastin and Basigin comprise a small family of neural cell adhesion proteins. Neuroplastin is an obligatory subunit of PMCA proteins, with PMCAs binding the transmembrane domain of Neuroplastin before being chaperoned to the plasma membrane.^21^^,^^32^ Moreover, Basigin has also been reported to be capable of facilitating membrane localization of PMCAs, although Basigin is reported to be less efficient at this than Neuroplastin.^21^ In the cochlea, PMCA2 and PMCA1 are both important for hair cell homeostasis through the extrusion of cytoplasmic *Ca*^*2+*^ ions into the extracellular fluid.^33^ Recently, we demonstrated that *Neuroplastin* genetically interacts with the *Cdh23*^*ahl*^ allele, with heterozygous *Neuroplastin* knockout mice only exhibiting hearing loss when maintained on a *Cdh23*^*ahl*^ genetic background (C57BL/6N) and not when maintained on a wild type *Cdh23* genetic background, i.e.,*.*, C57BL/6N-repair.^7^ We showed that loss of Neuroplastin led to a reduction in membrane localization of PMCAs, and as such the auditory phenotype was a result of the previously described genetic interaction between *Cdh23* and *Atp2b2*^34^*,* which encodes PMCA2.

In this present study, the finding that *Emb*^*tm1b/tm1b*^ mice exhibit hearing loss only when maintained on a *Cdh23*^*ahl*^ genetic background (C57BL/6N) mirrors what we have previously reported for heterozygous *Neuroplastin* knockout mice.^7^ Thus, we investigated whether Embigin, similar to Neuroplastin, interacts with PMCAs acting as a chaperone for their localization to the plasma membrane. However, unlike in the case of Neuroplastin, we could find no evidence of direct interaction between Embigin and any of the four PMCA proteins using co-immunoprecipitation of cochlear lysates (Figure 5C). This finding is consistent with a recent report where brain lysates were used.^21^ Moreover, we could find no evidence of reduced membrane localization of PMCA proteins in *Emb*^*tm1b/tm1b*^ cochlear hair cells (Figures 5A and 5B).

### Co-expression of *Emb*^*tm1b*^ and *Cdh23*^*ahl*^ leads to perinatal sub-viability

When maintained on the standard C57BL/6N background, it became apparent when intercrossing *Emb*^*+/tm1b*^ mice that *Emb*^*tm1b/tm1b*^ offspring were sub-viable. When genotyped at around PND14 just 12.8% of mice were recorded as homozygous, which is significantly less than the 25% expected by Mendelian ratios (Figure 6). Given there was no evidence for increased mortality between birth and PND14, it was presumed that *Emb*^*tm1b/tm1b*^ mice must be lost during embryonic development. To investigate the cause of sub-viability, we first harvested and genotyped embryos at E18.5. At this late gestational age, the embryo genotypes were obtained at the expected Mendelian ratios, suggesting that around half of the *Emb*^*tm1b/tm1b*^ mice die in the period either immediately before, during or soon after birth. Further investigation of the embryos utilizing microCT identified that a substantial proportion of the homozygous embryos had notable anatomical defects, including narrowing of the brain ventricles and cardiac interventricular septum defects.

In stark contrast to these findings, *Emb*^*tm1b/tm1b*^ mice maintained on the repaired C57BL/6N.*Cdh23*^*753A>G*^ background were recorded at the expected Mendelian ratios, when genotyped at ∼ PND14, and thus were not considered sub-viable. Furthermore, embryonic abnormalities at E18.5 were much less prevalent in *Emb*^*tm1b/tm1b*^ mice when maintained on the repaired background, suggesting that *Embigin* is interacting with *Cdh23* in additional organ systems outside of the auditory system.

Narrowing of brain ventricles has been previously observed as a co-morbidity in other deafness mouse mutants, *e.g*., *Aquaporin-4* (*Aqp4*)^35^ and *solute carrier family 4, sodium bicarbonate transporter, member 10* (*Slc4a10*).^36^ In the case of *Aqp4*, which is expressed in Hensen’s cells, Claudius cells, and inner sulcus cells as well as fibrocytes, loss of Aquaporin-4 water channels leads to profound deafness in mice by 4–5 weeks of age.^37^ Whereas in the case of *Slc4a10*, null mice were found to exhibit an early onset progressive hearing loss characterized by a reduced endocochlear potential. Consistent with this, Slc4a10 is expressed exclusively in type I, II, IV, and V fibrocytes of the murine cochlear lateral wall.^38^ Interestingly, in the case of *Slc4a10*, the early onset progressive deafness phenotype is reported in a knockout mutant maintained on a congenic C57BL/6J background.^38^ Whereas, in a different *Slc4a10* mutant (*Slc4a10*^*trombone*^) maintained on a mixed C57BL/6J; C3H.*Pde6b+* background, in which *Cdh23*^*ahl*^ was confirmed absent, a late-onset, mild hearing loss phenotype was instead observed (≥9-month of age).^39^ While these are two distinct *Slc4a10* mouse mutants, the difference in the onset and severity of their respective auditory phenotypes may be due to the presence/absence of the *Cdh23*^*ahl*^ allele. The finding of a late-onset mild hearing loss phenotype (≥9-month of age) in the *trombone* mutant also suggests that *Emb*^*tm1b/tm1b*^ mutant mice maintained on the repaired C57BL/6N.*Cdh23*^*c.753A>G*^ (*Cdh23*^*+*^) background may go on to exhibit an auditory deficit if aged beyond the 32-week reported here.

Outside of the cochlea, both *Aqp4* and *Slc4a10* are expressed in the epithelial cells of the choroid plexus and are thought to contribute to the release and homeostasis of cerebrospinal fluid,^35^^,^^36^ which is likely the primary cause of the reduction in ventricle size in these mutants. Data from the Allen Brain atlas shows that *Emb* mRNA is also strongly expressed in the choroid plexus epithelium (https://mouse.brain-map.org/experiment/show/73930836). However, while *Emb* transcripts are found in the choroid plexus, the same is not true for *Cdh23* (https://mouse.brain-map.org/experiment/show/7228380). Based on the expression patterns of *Cdh23* and *Emb* in the cochlea and brain, the genetic interaction reported here is unlikely to be due to a physical interaction between the two encoded proteins. Instead, loss of Embigin in cochlear support cells and/or the lateral wall may lead to an alteration in the properties of the endolymph that bathes the hair cell stereocilia, and this may cause an issue for hair cells expressing the hypomorphic *Cdh23*^*ahl*^ allele, but not those expressing wild type *Cdh23*. Additional studies are required to test this hypothesis, including the recording of endocochlear potentials.

### Implications for genetic research

While the functional requirement of Embigin within the mammalian auditory system remains to be fully elucidated, it is clear that Embigin has an important role. However, our data suggest that it is not an essential hearing gene, but instead loss of Embigin might only cause a hearing phenotype in an already sensitized system e.g., when the *Cdh23*^*ahl*^ allele is present.

The striking reversal of both the auditory and sub-viable phenotypes following the correction of the *Cdh23*^*ahl*^ allele also highlights the importance of mouse strain selection in genetic studies, particularly those investigating neurological and cardiac disorders. In fact, a number of inbred mouse strains carry the *Cdh23*^*ahl*^ allele, which in addition to the very commonly used C57BL/6 strains also include BALB/cBy, DBA/1J, and DBA/2J (http://www.informatics.jax.org/allele/MGI:3028349). Moreover, our findings also highlight the importance of understanding and controlling the genetic background of mouse mutants maintained on a mixed genetic background, as the presence or absence of strain-specific alleles in combination with the mutation of interest could lead to greater variation in phenotype expressivity, and subsequently the requirement to breed and phenotype larger cohorts to gain statistical significance.

Finally, in spite of *Cdh23* being expressed in a wide array of different tissues (https://www.proteinatlas.org/ENSG00000107736-CDH23/tissue), it is largely still considered a cochlear gene, with the primary function of the encoded protein being to form (together with PCDH15) stereocilia tip links that are essential for the mechanoelectrical transduction of soundwave-induced vibrations. As such, although Cadherin 23 has also been shown to play a role in the development of interneurons of the auditory cortex,^40^ the *ahl* allele has been considered relatively “inert”, unless studying hearing or using an auditory cue as part of a phenotyping paradigm. However, our study strongly suggests the *Cdh23*^*ahl*^ allele has important effects in organs outside of the auditory system, particularly during embryonic development. Indeed, our data would suggest that the use of C57BL/6N as the base strain for the generation of knockout mutants within the IMPC means that this program could be considered a sensitized screen. For instance, like *Embigin*, some of the IMPC knockout lines reported to have increased hearing thresholds may not exhibit hearing loss if maintained on a different inbred strain that does not harbor the *Cdh23*^*ahl*^ allele. Moreover, the consortium report that through observing the number of homozygous loss-of-function offspring generated from an intercross between heterozygous parents it has allowed them to categorize ∼10% of gene knockouts as sub-viable.^41^ It would be interesting to determine how many of these knockout mutant lines would still exhibit sub-viability if they were maintained on an inbred strain that does not harbor the *Cdh23*^*ahl*^ allele, such as the co-isogenic C57BL/6N.*Cdh23*^*c.753A>G*^ used in this study.

### Limitations of the study

In the present study, we were unable to identify a mechanism for the accelerated hearing deficit observed in the C57BL/6N;*Embigin*^*tm1b/tm1b*^ mice. However, given the expression of Embigin in the lateral supporting cells and the presentation of the phenotype, we hypothesize that a dysfunction in the maintenance of the endocochlear potential in these adult mutant mice may be the cause. Recording of their endocochlear potential would be required to confirm this, unfortunately we are not currently equipped to undertake these measurements.

The present study shows the integrity and morphology of OHC stereocilia in C57BL/6N;*Embigin*^*tm1b/tm1b*^ mice are unaffected at 6-week of age. To further support these findings, we had planned to age our phenotyping cohort to 24-week and undertake ABR and DPOAE recordings, and collect cochlear samples for SEM imaging. Unfortunately, due to restrictions on movement imposed in response to the COVID-19 pandemic we were not able to perform these studies at this time point.

Lastly, we did not investigate whether the surviving adult C57BL/6N;*Embigin*^*tm1b/tm1b*^ mice exhibit cardiac septum defects. Given we find 100% penetrance of cardiac ventricle defects in C57BL/6N;*Embigin*^*tm1b/tm1b*^ embryos at E18.5 this suggests that some are able to survive into adulthood, which if confirmed would make these mice a ventricular septal defect model.

## STAR★Methods

### Key resources table

**Table:** 

REAGENT or RESOURCE	SOURCE	IDENTIFIER
**Antibodies**		

Rat anti-Embigin G7.43.1	Invitrogen	Cat# 14-5839-81, RRID: AB_2016582
Rabbit anti-Embigin	Novus	Cat# NBP2-24689, RRID:AB_2941092
Mouse anti-PMCA 5F10	Thermo Fisher Scientific	Cat# MA3-914, RRID:AB_2061566
Mouse (IgG1) anti-Ctbp	BD	Cat# 612044, RRID: AB_399431
Mouse (IgG2a) anti-GluR2	Millipore	Cat# MAB397, RRID: AB_2113875
Rabbit anti-Myosin7a	Proteus Biosciences	Cat# 25–6790, RRID: AB_10015251
Mouse anti-β-actin	Proteintech	Cat# 60008-1-Ig, RRID:AB_2289225
Mouse anti-β-Tubulin III	Sigma-Aldrich	Cat# 25–6790, RRID: AB_10015251
Alexa Fluor 647 donkey anti-rabbit	Thermo Fisher Scientific	Cat# A-21244, RRID:AB_2535812
Alexa Fluor 594 donkey anti-Rat	Thermo Fisher Scientific	Cat# A-21209, RRID: AB_2535795
Alexa Fluor 488 donkey anti-mouse	Thermo Fisher Scientific	Cat# A-21202, RRID:AB_141607
Alexa Fluor 568 goat anti- mouse IgG1	Thermo Fisher Scientific	Cat# A21124, RRID:AB_141611
Alexa Fluor 488 goat anti- mouse IgG2a	Thermo Fisher Scientific	Cat# A21131, RRID:AB_2535771
Dylight 405 AffiniPure donkey anti-rabbit IgG	Jackson Immuno Research Labs	Cat# 711-475-152, RRID:AB_2340616
HRP-conjugated goat anti-Rat	Invitrogen	Cat# 31470, RRID:AB_228356
Donkey anti-mouse IRDye 800CW	LI-COR Biosciences	Cat# 926–32212, RRID:AB_621847
Goat anti-rat IRDye 680RD	LI-COR Biosciences	Cat# 926–68076, RRID:AB_10956590

**Experimental models: Organisms/strains**		

C57BL/6N-*Emb*^*tm1b(KOMP)Wtsi*^	Mutant Mouse Resource and Research Center (MMRRC)	RRID:MGI:5767650
C57BL/6NTac	Mary Lyon Center at Harwell	RRID:IMSR_TAC:B6
*Cdh23*^*ahl+em3H*^*/Cdh23*^*753A>G*^	Mary Lyon Center at Harwell	RRID:MGI:5749861

**Chemicals, peptides, and recombinant proteins**		

PNGase F	NEB	Cat# P0704S
X-Gal	Thermo Scientific	Cat# R0404
Pro-Long Gold Antifade Mountant	Invitrogen	Cat# P36930
Dynabeads™ Protein G	Invitrogen	Cat# 10003D

**Critical commercial assays**		

High-Capacity RNA-to-cDNA™ Kit	Applied Biosystems	Cat# 4387406
*Emb* TaqMan Assay	Thermo Fisher Scientific	assay ID Mm00515881_m1
*Hprt* TaqMan Assay	Thermo Fisher Scientific	assay ID Mm03024075_m1

**Deposited data**		

Mouse Brain Atlas	Allen Brain Atlas	https://mouse.brain-map.org/
CDH23 Expression map	Human Protein Atlas	https://www.proteinatlas.org
Ensembl (release 109) Mouse reference genome GRCm39	ensembl.org	https://www.ensembl.org

**Software and algorithms**		

BiosigRZ (version 5.7.1)	Tucker Davies Technology	https://www.tdt.com/component/biosigrz-abr-dpoae-software/
GraphPad Prism 9.0	GraphPad by Dotmatics	https://www.graphpad.com/
NDP.view2 Image viewer	Hamamatsu	Cat# U12388-01
ZEN Microscopy Software	Zeiss	https://www.zeiss.com/microscopy/en/products/software/zeiss-zen.html
Fiji	ImageJ	https://imagej.net/software/fiji/
NRecon	Bruker Micro-CT Software	https://blue-scientific.com/bruker-micro-ct/micro-ct-software/

**Other**		

TDT RZ6 System 3	Tucker Davies Technology	https://www.tdt.com/products/
NanoZoomer Slide Scanner	Hamamatsu	NanoZoomer 2.0 -RS, discontinued.
Confocal Microscope	Zeiss	Model: LSM 710
Scanning Electron Microscope	JEOL	JSM 6010LV
Chemiluminescent imager (Western blot)	UVP	ChemiDoc-It
Fluorescent imager (Western blot)	LI-COR	Odyssy DLx Imager
X-ray micro-CT system	Bruker	Skyscan 1172 scanner

### Resource availability

#### Lead contact

Further information and requests for resources and reagents should be directed to, and will be fulfilled by the lead contact, Dr Michael R Bowl (m.bowl@ucl.ac.uk).

#### Materials availability

This study did not generate any new materials. All animal models used are available through the MMRRC repository.

### Experimental model and study participant details

#### *In vivo* animal studies

All animal studies were licensed by the Home Office under the Animals (Scientific Procedures) Act 1986, United Kingdom, and additionally approved by Institutional Ethical Review Committees (PBF9BD884). Mice were group housed under a 12-h light/12-h dark cycle and allowed access to food and water *ad libitum*. The mouse strain used for this research project, *Emb*^*tm1b(KOMP)Wtsi*^ (hereafter called *Emb*^*tm1b*^, RRID:MGI:5767650) was obtained from the Mutant Mouse Resource and Research Center (MMRRC) at the University of California at Davis, an NIH-funded strain repository, and was donated to the MMRRC by The KOMP Repository, University of California, Davis; UC Davis Mouse Biology Program. This line was generated on a C57BL/6N background as part of the International Mouse Phenotyping Consortium and produced through Cre-mediated conversion of the ‘knockout-first’ *tm1a* allele, which was achieved through the breeding heterozygous *tm1a* mice with a ubiquitous Cre deleter mouse line for recombination of the *LoxP* sites. In the converted *tm1b* allele, exon 5 (ENSMUSE00000436647) of the *Embigin* gene is deleted, leaving the *lacZ* reporter cassette (Figure 1A), containing a splice acceptor that subsumes normal splicing. Thus, no functional protein is expected to be produced from the *Emb*^*tm1b*^ allele. All mutant mouse models used were maintained on a C57BL/6NTac background (RRID:IMSR_TAC:b6) or were crossed for two generations onto a C57BL/6NTac;*Cdh23*-repaired background (RRID:MGI:5749861) in which both alleles of the hypomorphic *Cdh23*^*ahl*^ allele present in the C57BL/6 background is corrected to wild type (*Cdh23*^*753A>G*^).^16^ No sex-based differences in phenotype were observed during the IMPC adult phenotyping pipeline, or this present study; as such, both male and female mice were used for all experiments. Ages of mice at the time of experiments are stated within the main text.

### Method details

#### Auditory brainstem response (ABR)

Analysis of hearing function by ABR was undertaken. Mice were anesthetised with an intraperitoneal (I.P.) injection of ketamine (100 mg mL^−1^ at 10% v/v) and xylazine (20 mg mL+ at 5% v/v) administered at a rate of 0.1 mL/10 g body mass. Animals were placed on a heated mat inside a sound-attenuated chamber (ETS-Lindgren), and electrodes were inserted sub-dermally; below the right pinna, into the muscle mass below the left ear, and at the cranial vertex. ABR responses were collected, amplified, and averaged using the TDT RZ6 System 3 hardware in conjunction with BioSigRZ (version 5.7.1) software (Tucker Davies Technology). Stimuli were delivered in the form of a 0.1 ms broadband click, or as a single frequency tone at 6, 12, 18, 24, and 30 kHz (5 ms duration, 1 ms rise and fall). All stimuli were presented in 5 dB falling steps from 90 dB SPL, and responses were averaged over 512 (tone) or 300 (click) repeats. Following ABR recordings, mice were either culled by cervical dislocation or recovered with an S.C. injection of atipamezole administered 1 mg kg^−1^ body mass. For the analysis of wave I, supra-threshold traces were further filtered between 400 and 3000 Hz to remove additional background noise. Amplitudes were calculated as the difference between the peak and valley. Latencies were calculated as the time from onset to the wave peak. Primary analysis of the recorded ABRs were performed with the BiosigRZ software, further statistical tests were performed using GraphPad Prism 9.0.

#### Distortion product otoacoustic emissions (DPOAEs)

To assess outer hair cell function *in vivo*, surgical anesthesia was induced by intraperitoneal injection of ketamine (100 mg mL^−1^ at 10% v/v), xylazine (20 mg mL^−1^ at 5% v/v) and acepromazine (2 mg mL^−1^ at 8% v/v), administered at a rate of 0.1 mL/10 g body mass. Once the required depth of anesthesia was confirmed by the lack of the pedal reflex, a section of pinna was removed to allow unobstructed access to the external auditory meatus. Mice were then placed on a heated mat inside a sound-attenuated chamber (ETS-Lindgren) and the DPOAE probe assembly was inserted into the ear canal using a pipette tip to aid in correct placement. DPOAE tests were performed using frequency-specific tone-burst stimuli at 6, 12, 18, 24, and 30 kHz with the TDT RZ6 System 3 hardware and BioSigRZ (version 5.7.1) software (Tucker Davis Technology). An ER10B+ low noise probe microphone (Etymotic Research) was used to measure the DPOAE near the tympanic membrane. Tone stimuli were presented via separate MF1 (Tucker Davis Technology) speakers, with f1 and f2 at a ratio of f2/f1 = 1.2 (L1 = 65 dB SPL, L2 = 55 dB SPL), centered around the frequencies of 6, 12, 18, 24, and 30 kHz. In-ear calibration was performed before each test. The f1 and f2 tones were presented continuously and a fast-Fourier transform was performed on the averaged response of 356 epochs (each approximately 21 ms). The level of the 2f1 − f2 DPOAE response was recorded and the noise floor was calculated by averaging the four frequency bins on either side of the 2f1 − f2 frequency. DPOAEs presented as the response above the average noise floor. Following DPOAE recordings, mice were culled by cervical dislocation.

#### Histology

Histological examination of the inner ear has been described previously in detail by Hardisty-Hughes et al.^42^ Briefly, to assess the gross morphology of the cochlea, mice were euthanised by cervical dislocation. Skinned and bisected heads were fixed by submersion in 10% neutral-buffered formaldehyde for >72 h at room temperature (20°C). Fixed samples were decalcified by submersion in 50 mL of D.F.B (decalcification solution), for 2 days at room temperature, before embedding in paraffin. 5 μm-thick sagittal sections were prepared and stained with H&E. Slides were digitally scanned using a Hamamatsu NanoZoomer slide scanner with 20× magnification and analyzed using NDP.view2 Image viewing software.

For the localisation of Embigin by X-Gal staining, animals were culled by cervical dislocation and inner ears were removed and fixed by perfusion at the round and oval window with 4% paraformaldehyde (PFA), followed by 1 h submersion fixation on ice. Whole cochleae were submerged in an X-Gal staining solution (1 mg/mL X-Gal, 5 mM Potassium Ferrocyanide, 5 mM Potassium Ferricyanide, 2 mM MgCl_2_, 0.02% (v/v) IGEPAL CA-630, 10% (v/v) sodium deoxycholate) for 20 h at 37°C, before washing and post-fixing in 4% PFA for 2 h at room temperature (20°C). Sections were cut from stained and fixed tissues decalcified for 72 h at 4°C in 3.5% EDTA, cryoprotected with 30% sucrose, and then embedded in OCT before cutting into 12 μm slices on a cryostat. Images were captured on a Zeiss Axio Observer Z1 in conjunction with Zeiss ZEN microscopy software.

#### Scanning electron microscopy

To assess the hair cell stereocilia morphology, the inner ears were removed into 2.5% glutaraldehyde in 0.1 M sodium phosphate buffer overnight at 4°C. Postfixation, ears were incubated in 4.3% EDTA at 4°C until sufficiently decalcified before being dissected to expose the organ of Corti. To preserve stereocilia structure, samples were processed with alternating 1% osmium tetroxide (O) and 1% thiocarbohydrazide (T) treatments (OTO processing) and then dehydrated in increasing concentrations of ethanol at 4°C. Samples were mounted on stubs and critical point dried with liquid CO_2_ before being sputter coated using a platinum target and visualised using a JEOL JSM 6010LV Scanning Electron Microscope.

#### MicroCT

Embryos were dissected into ice-cold phosphate buffered saline (PBS) and exsanguinated by severing the umbilical vessels. After washing in PBS, embryos were fixed by immersion in 4% PFA (in PBS) for 7 days at 4°C, before storing in 1% PFA at 4°C.

Embryos were contrasted by immersion in 50% Lugol’s solution for 2-week, protected from light, and solution exchanged for fresh every 2 days. After contrasting, samples were washed in ddH_2_O for at least 1 h before embedding in an acrylic mount in 1% agarose dissolved in ddH_2_O and allowed to set for at least 2 h.

MicroCT datasets were acquired using a Skyscan 1172 scanner (Bruker). For embryos, scans were carried out with an X-ray source set to 80 kV and using a 0.5 mm aluminum filter, two averaged projections were acquired every 0.25° through a total rotation of 180° at 5 μm/pixel resolution. NRecon (Bruker) was used for 3D reconstruction and embryo scans were further cropped and scaled to 28 μm isotropic voxels using the HARP software.^26^

#### Immunohistochemistry

To assess protein localisation in the cochlea, animals were culled by cervical dislocation and inner ears were removed and fixed by perfusion at the round and oval window with 4% PFA, followed by 1 h submersion fixation on ice. Ears were finely dissected to expose the sensory epithelium. For synaptic labeling studies, cochleae were permeabilised using PBS +0.3% Triton X-100, blocked in 5% goat serum, and incubated at 37°C overnight (16–18 h) with mouse (IgG1) anti-Ctbp2 (1:200, BD, Cat# 612044, RRID: AB_399431), mouse (IgG2a) anti-GluR2 (1:200, Millipore, Cat# MAB397, RRID: AB_2113875), and rabbit anti-Myosin7a (1:200, Proteus Biosciences, Cat# 25–6790, RRID: AB_10015251). Secondary antibodies were applied for 2 × 1 h at 37°C; Alexa Fluor 568 goat anti-mouse IgG1 (1:1000, Thermo Fisher Scientific, Cat# A21124, RRID:AB_141611), Alexa Fluor 488 goat anti-mouse IgG2a (1:1000, Thermo Fisher Scientific, Cat# A21131, RRID:AB_2535771), and Dylight 405 AffiniPure donkey anti-rabbit IgG (1:1000, Jackson Immuno Research Labs, Cat# 711-475-152, RRID:AB_2340616). For all other immunohistochemistry studies, cochleae were permeabilised with PBS +0.3% Triton X-100, blocked in 5% donkey serum +1% BSA, and incubated with primary antibodies overnight (16–18 h) at 4°C. Primary antibodies: rat anti-Embigin G7.43.1 (1:50, Invitrogen, Cat# 14-5839-81, RRID: AB_2016582), mouse anti-PMCA 5F10 (1:200, Thermo Fisher Scientific, Cat# MA3-914, RRID:AB_2061566), rabbit anti-Myosin7a (1:500, Proteus Biosciences, Cat# 25–6790, RRID: AB_10015251), and mouse anti-β-Tubulin III (1:200, Sigma-Aldrich Cat# T8578, RRID:AB_1841228). Secondary antibodies were applied for 2 h at room temperature (20°C); Alexa Fluor 594 donkey anti-Rat (1:500, Thermo Fisher Scientific, Cat# A-21209, RRID: AB_2535795) Alexa Fluor 488 donkey anti-mouse (1:500, Thermo Fisher Scientific, Cat# A-21202, RRID:AB_141607), and Alexa Fluor 647 donkey anti-rabbit (1:500, Thermo Fisher Scientific, Cat# A-21244, RRID:AB_2535812). Slides were mounted for examination under a confocal microscope with Pro-Long Gold Antifade mountant. Samples were visualised using a Zeiss LSM 710 with or without Airyscan detector under either 20× magnification or 63× oil magnification (synapses). Images were processed using the Zeiss Zen microscopy software and Fiji.

#### Western Blot

Whole cochlea lysates were prepared using an NP-40 lysis buffer (150 mM NaCl, 50 mM Tris, 1% NP-40). Glycosylation was removed from denatured extracted lysates using PNGase F (NEB, Cat# P0704S) as per the manufacturer’s protocol, and protein (25 μg) was separated with SDS/PAGE and transferred onto 0.45 μm Nitrocellulose membranes (Invitrogen, Cat# LC2001). Membranes were blocked with 5% milk diluted in TBS +0.1% Tween 20 and room temperature for 1 h, then probed with a rat anti-Embigin Monoclonal Antibody G7.43.1 (1:200, Invitrogen, Cat# 14-5839-81, RRID: AB_2016582) or a rabbit anti-Embigin polyclonal antibody (1:100, Novus Biologicals, Cat# NBP2-24689, RRID:AB_2941092) overnight (16–18 h) at 4°C. Equal loading was confirmed with antibodies against β-actin (1:5000, Proteintech, Cat# 60008-1-Ig, RRID:AB_2289225). For detection, membranes were incubated with an HRP-conjugated goat anti-Rat antibody (1:1000, Invitrogen, Cat# 31470, RRID:AB_228356) and imaged with a UVP ChemiDoc-It imaging system with a BioChemi HR camera.

For the immunoprecipitation of PMCA-associated proteins, 10 cochleae (5 mice) were pooled into a single sample and lysed using an NP-40 lysis buffer (150 mM NaCl, 50 mM Tris, 1% NP-40). Total protein (1 mg) was pre-cleared with Dynabeads Protein G (Invitrogen, Cat# 10003D) for 1 h at 4°C, then incubated with mouse anti-PMCA 5F10 (1 mg/mL, Thermo Fisher Scientific, Cat# MA3-914, RRID:AB_2061566) overnight (16–18 h) at 4°C. For the negative control, 1 mg of total protein was pre-cleared and then incubated without antibodies overnight. The antibody and its bound proteins were isolated using Dynabeads Protein G, and the isolated protein was eluted from the beads before being separated with SDS/PAGE and transferred onto 0.45 μm Nitrocellulose membranes (InvitrogenTM, Cat# LC2001). Membranes were blocked with 5% milk for 1 h at room temperature, then probed with a rat Embigin Monoclonal Antibody G7.43.1 (1:200, Invitrogen, Cat# 14-5839-81, RRID: AB_2016582) and mouse anti-PMCA 5F10 (1:1000, Thermo Fisher Scientific, Cat# MA3-914, RRID:AB_2061566) overnight (16–18 h) at 4°C. For detection, membranes were incubated for 2 h at room temperature with donkey anti-mouse IRDye 800CW (1:10000, LI-COR Biosciences, Cat# 926–32212, RRID:AB_621847) and goat anti-rat IRDye 680RD (1:10000, LI-COR Biosciences, Cat# 926–68076, RRID:AB_10956590), and visualised using the Odyssey DLx Imager (LI-COR). Application of primary and secondary antibodies were performed independently on the same membrane to rule out cross-reactivity between mouse- and rat- IgGs.

#### cDNA synthesis and quantitative RT-PCR (qPCR)

200 ng of total RNA was used for cDNA synthesis. cDNA synthesis was performed using High-Capacity RNA-to-cDNA Kit (Applied Biosystems) according to manufacturer’s protocol. qPCR amplification of *Emb* gene was performed using a TaqMan assay (assay ID Mm00515881_m1, Thermo Fisher Scientific), in the QuantStudio 7 Pro Real-Time PCR System (Thermo Fisher Scientific). Hypoxanthine guanine phosphoribosyl transferase (*Hprt*) was used as housekeeping gene (assay ID Mm03024075_m1). Technical triplicates were performed to reduce experimental errors. Additionally, negative controls with only PCR-grade water as a template were run to rule out possible contaminations. Statistical analyses were performed using Student’s *t* test. A standard curve was performed using a serial dilution of 0.002–200 ng target cDNA per reaction. Duplicates were performed for each standard dilution.

### Quantification and statistical analysis

Mean values are quoted in text and figures as mean ± standard deviation (SD), except for ABR data which is displayed as median ± interquartile range (IQR). Statistical comparisons of means were made by analysis of variance (one-way or two-way ANOVA) followed by a suitable post-test, or by Student’s two-tailed t-test with or without correction for multiple comparisons. p values less than 0.05 were considered statistically significant. Data analysis was performed using GraphPad Prism 9.0. ABR thresholds were second scored by an independent researcher blinded to genotype and overall trends and levels of significance were confirmed to match. For the analysis of ABR thresholds where true values were out of the range of our equipment, thresholds were given a value of 95 dB SPL and marked as “no response” (NR).

## Data Availability

• All data reported in this paper will be shared by the lead contact upon request. • This paper does not report original code. • Any additional information required to re-analyse the data reported in this paper is available from the lead contact upon request.

## References

[1] Bowl M.R., Brown S.D.M. (2018). Genetic landscape of auditory dysfunction. Hum. Mol. Genet..

[2] Yamasoba T., Lin F.R., Someya S., Kashio A., Sakamoto T., Kondo K. (2013). Current concepts in age-related hearing loss: epidemiology and mechanistic pathways. Hear. Res..

[3] Bowl M.R., Simon M.M., Ingham N.J., Greenaway S., Santos L., Cater H., Taylor S., Mason J., Kurbatova N., Pearson S. (2017). A large scale hearing loss screen reveals an extensive unexplored genetic landscape for auditory dysfunction. Nat. Commun..

[4] Ohlemiller K.K., Jones S.M., Johnson K.R. (2016). Application of Mouse Models to Research in Hearing and Balance. J. Assoc. Res. Otolaryngol..

[5] Carrott L., Bowl M.R., Aguilar C., Johnson S.L., Chessum L., West M., Morse S., Dorning J., Smart E., Hardisty-Hughes R. (2016). Absence of Neuroplastin-65 Affects Synaptogenesis in Mouse Inner Hair Cells and Causes Profound Hearing Loss. J. Neurosci..

[6] Zeng W.Z., Grillet N., Dewey J.B., Trouillet A., Krey J.F., Barr-Gillespie P.G., Oghalai J.S., Müller U. (2016). Neuroplastin Isoform Np55 Is Expressed in the Stereocilia of Outer Hair Cells and Required for Normal Outer Hair Cell Function. J. Neurosci..

[7] Newton S., Kong F., Carlton A.J., Aguilar C., Parker A., Codner G.F., Teboul L., Wells S., Brown S.D.M., Marcotti W., Bowl M.R. (2022). Neuroplastin genetically interacts with Cadherin 23 and the encoded isoform Np55 is sufficient for cochlear hair cell function and hearing. PLoS Genet..

[8] Ozawa M., Huang R.P., Furukawa T., Muramatsu T. (1988). A teratocarcinoma glycoprotein carrying a developmentally regulated carbohydrate marker is a member of the immunoglobulin gene superfamily. J. Biol. Chem..

[9] Huang R.P., Ozawa M., Kadomatsu K., Muramatsu T. (1990). Developmentally regulated expression of embigin, a member of the immunoglobulin superfamily found in embryonal carcinoma cells. Differentiation..

[10] Kazmierczak P., Sakaguchi H., Tokita J., Wilson-Kubalek E.M., Milligan R.A., Müller U., Kachar B. (2007). Cadherin 23 and protocadherin 15 interact to form tip-link filaments in sensory hair cells. Nature.

[11] Noben-Trauth K., Zheng Q.Y., Johnson K.R. (2003). Association of cadherin 23 with polygenic inheritance and genetic modification of sensorineural hearing loss. Nat. Genet..

[12] Hequembourg S., Liberman M.C. (2001). Spiral ligament pathology: a major aspect of age-related cochlear degeneration in C57BL/6 mice. J. Assoc. Res. Otolaryngol..

[13] Keithley E.M., Canto C., Zheng Q.Y., Fischel-Ghodsian N., Johnson K.R. (2004). Age-related hearing loss and the ahl locus in mice. Hear. Res..

[14] Jeng J.Y., Johnson S.L., Carlton A.J., De Tomasi L., Goodyear R.J., De Faveri F., Furness D.N., Wells S., Brown S.D.M., Holley M.C. (2020). Age-related changes in the biophysical and morphological characteristics of mouse cochlear outer hair cells. J. Physiol..

[15] Jeng J.Y., Carlton A.J., Johnson S.L., Brown S.D.M., Holley M.C., Bowl M.R., Marcotti W. (2021). Biophysical and morphological changes in inner hair cells and their efferent innervation in the ageing mouse cochlea. J. Physiol..

[16] Mianne J., Chessum L., Kumar S., Aguilar C., Codner G., Hutchison M., Parker A., Mallon A.M., Wells S., Simon M.M. (2016). Correction of the auditory phenotype in C57BL/6N mice via CRISPR/Cas9-mediated homology directed repair. Genome Med..

[17] Yasuda S.P., Seki Y., Suzuki S., Ohshiba Y., Hou X., Matsuoka K., Wada K., Shitara H., Miyasaka Y., Kikkawa Y. (2020). c.753A>G genome editing of a Cdh23(ahl) allele delays age-related hearing loss and degeneration of cochlear hair cells in C57BL/6J mice. Hear. Res..

[18] Kane K.L., Longo-Guess C.M., Gagnon L.H., Ding D., Salvi R.J., Johnson K.R. (2012). Genetic background effects on age-related hearing loss associated with Cdh23 variants in mice. Hear. Res..

[19] Herrera-Molina R., Mlinac-Jerkovic K., Ilic K., Stöber F., Vemula S.K., Sandoval M., Milosevic N.J., Simic G., Smalla K.H., Goldschmidt J. (2017). Neuroplastin deletion in glutamatergic neurons impairs selective brain functions and calcium regulation: implication for cognitive deterioration. Sci. Rep..

[20] Korthals M., Langnaese K., Smalla K.H., Kähne T., Herrera-Molina R., Handschuh J., Lehmann A.C., Mamula D., Naumann M., Seidenbecher C. (2017). A complex of Neuroplastin and Plasma Membrane Ca(2+) ATPase controls T cell activation. Sci. Rep..

[21] Schmidt N., Kollewe A., Constantin C.E., Henrich S., Ritzau-Jost A., Bildl W., Saalbach A., Hallermann S., Kulik A., Fakler B., Schulte U. (2017). Neuroplastin and Basigin Are Essential Auxiliary Subunits of Plasma Membrane Ca(2+)-ATPases and Key Regulators of Ca(2+) Clearance. Neuron.

[22] Lin X., Brunk M.G.K., Yuanxiang P., Curran A.W., Zhang E., Stöber F., Goldschmidt J., Gundelfinger E.D., Vollmer M., Happel M.F.K. (2021). Neuroplastin expression is essential for hearing and hair cell PMCA expression. Brain Struct. Funct..

[23] Muramatsu T. (2016). Basigin (CD147), a multifunctional transmembrane glycoprotein with various binding partners. J. Biochem..

[24] Brajon S., Morello G.M., Capas-Peneda S., Hultgren J., Gilbert C., Olsson A. (2021). All the Pups We Cannot See: Cannibalism Masks Perinatal Death in Laboratory Mouse Breeding but Infanticide Is Rare. Animals (Basel).

[25] Dickinson M.E., Flenniken A.M., Ji X., Teboul L., Wong M.D., White J.K., Meehan T.F., Weninger W.J., Westerberg H., Adissu H. (2016). High-throughput discovery of novel developmental phenotypes. Nature.

[26] Brown J.M., Horner N.R., Lawson T.N., Fiegel T., Greenaway S., Morgan H., Ring N., Santos L., Sneddon D., Teboul L. (2018). A bioimage informatics platform for high-throughput embryo phenotyping. Brief. Bioinform..

[27] Kikuchi T., Kimura R.S., Paul D.L., Adams J.C. (1995). Gap junctions in the rat cochlea: immunohistochemical and ultrastructural analysis. Anat. Embryol..

[28] Ovens M.J., Manoharan C., Wilson M.C., Murray C.M., Halestrap A.P. (2010). The inhibition of monocarboxylate transporter 2 (MCT2) by AR-C155858 is modulated by the associated ancillary protein. Biochem. J..

[29] Wilson M.C., Meredith D., Fox J.E.M., Manoharan C., Davies A.J., Halestrap A.P. (2005). Basigin (CD147) is the target for organomercurial inhibition of monocarboxylate transporter isoforms 1 and 4: the ancillary protein for the insensitive MCT2 is EMBIGIN (gp70). J. Biol. Chem..

[30] Xu B., Zhang M., Zhang B., Chi W., Ma X., Zhang W., Dong M., Sheng L., Zhang Y., Jiao W. (2022). Embigin facilitates monocarboxylate transporter 1 localization to the plasma membrane and transition to a decoupling state. Cell Rep..

[31] Fisel P., Schaeffeler E., Schwab M. (2018). Clinical and Functional Relevance of the Monocarboxylate Transporter Family in Disease Pathophysiology and Drug Therapy. Clin. Transl. Sci..

[32] Gong D., Chi X., Ren K., Huang G., Zhou G., Yan N., Lei J., Zhou Q. (2018). Structure of the human plasma membrane Ca(2+)-ATPase 1 in complex with its obligatory subunit neuroplastin. Nat. Commun..

[33] Boyer C., Art J.J., Dechesne C.J., Lehouelleur J., Vautrin J., Sans A. (2001). Contribution of the plasmalemma to Ca2+ homeostasis in hair cells. J. Neurosci..

[34] Watson C.J., Tempel B.L. (2013). A new Atp2b2 deafwaddler allele, dfw(i5), interacts strongly with Cdh23 and other auditory modifiers. Hear. Res..

[35] Trillo-Contreras J.L., Toledo-Aral J.J., Echevarria M., Villadiego J. (2019). AQP1 and AQP4 Contribution to Cerebrospinal Fluid Homeostasis. Cells.

[36] Jacobs S., Ruusuvuori E., Sipilä S.T., Haapanen A., Damkier H.H., Kurth I., Hentschke M., Schweizer M., Rudhard Y., Laatikainen L.M. (2008). Mice with targeted Slc4a10 gene disruption have small brain ventricles and show reduced neuronal excitability. Proc. Natl. Acad. Sci. USA.

[37] Li J., Verkman A.S. (2001). Impaired hearing in mice lacking aquaporin-4 water channels. J. Biol. Chem..

[38] Huebner A.K., Maier H., Maul A., Nietzsche S., Herrmann T., Praetorius J., Hübner C.A. (2019). Early Hearing Loss upon Disruption of Slc4a10 in C57BL/6 Mice. J. Assoc. Res. Otolaryngol..

[39] Potter P.K., Bowl M.R., Jeyarajan P., Wisby L., Blease A., Goldsworthy M.E., Simon M.M., Greenaway S., Michel V., Barnard A. (2016). Novel gene function revealed by mouse mutagenesis screens for models of age-related disease. Nat. Commun..

[40] Libé-Philippot B., Michel V., Boutet de Monvel J., Le Gal S., Dupont T., Avan P., Métin C., Michalski N., Petit C. (2017). Auditory cortex interneuron development requires cadherins operating hair-cell mechanoelectrical transduction. Proc. Natl. Acad. Sci. USA.

[41] Cacheiro P., Muñoz-Fuentes V., Murray S.A., Dickinson M.E., Bucan M., Nutter L.M.J., Peterson K.A., Haselimashhadi H., Flenniken A.M., Morgan H. (2020). Human and mouse essentiality screens as a resource for disease gene discovery. Nat. Commun..

[42] Hardisty-Hughes R.E., Parker A., Brown S.D.M. (2010). A hearing and vestibular phenotyping pipeline to identify mouse mutants with hearing impairment. Nat. Protoc..

