# Supplementary material for: Absence of Embigin accelerates hearing loss and causes sub-viability, brain and heart defects in C57BL/6N mice due to interaction with Cdh23ahl
Source: iScience. 2023 Sep 26;26(10):108056. doi: 10.1016/j.isci.2023.108056 (PMC10579432; doi:10.1016/j.isci.2023.108056)
Supplement: Document S1. Figures S1–S5 [file mmc1.pdf]

iScience, Volume *26*

Supplemental information

Absence of Embigin accelerates hearing loss and causes sub-viability, brain and heart defects in C57BL/6N mice due to interaction with *Cdh23*^{ahl}

Sherylanne Newton, Carlos Aguilar, Rosie K. Bunton-Stasyshyn, Marisa Flook, Michelle Stewart, Walter Marcotti, Steve Brown, and Michael R. Bowl

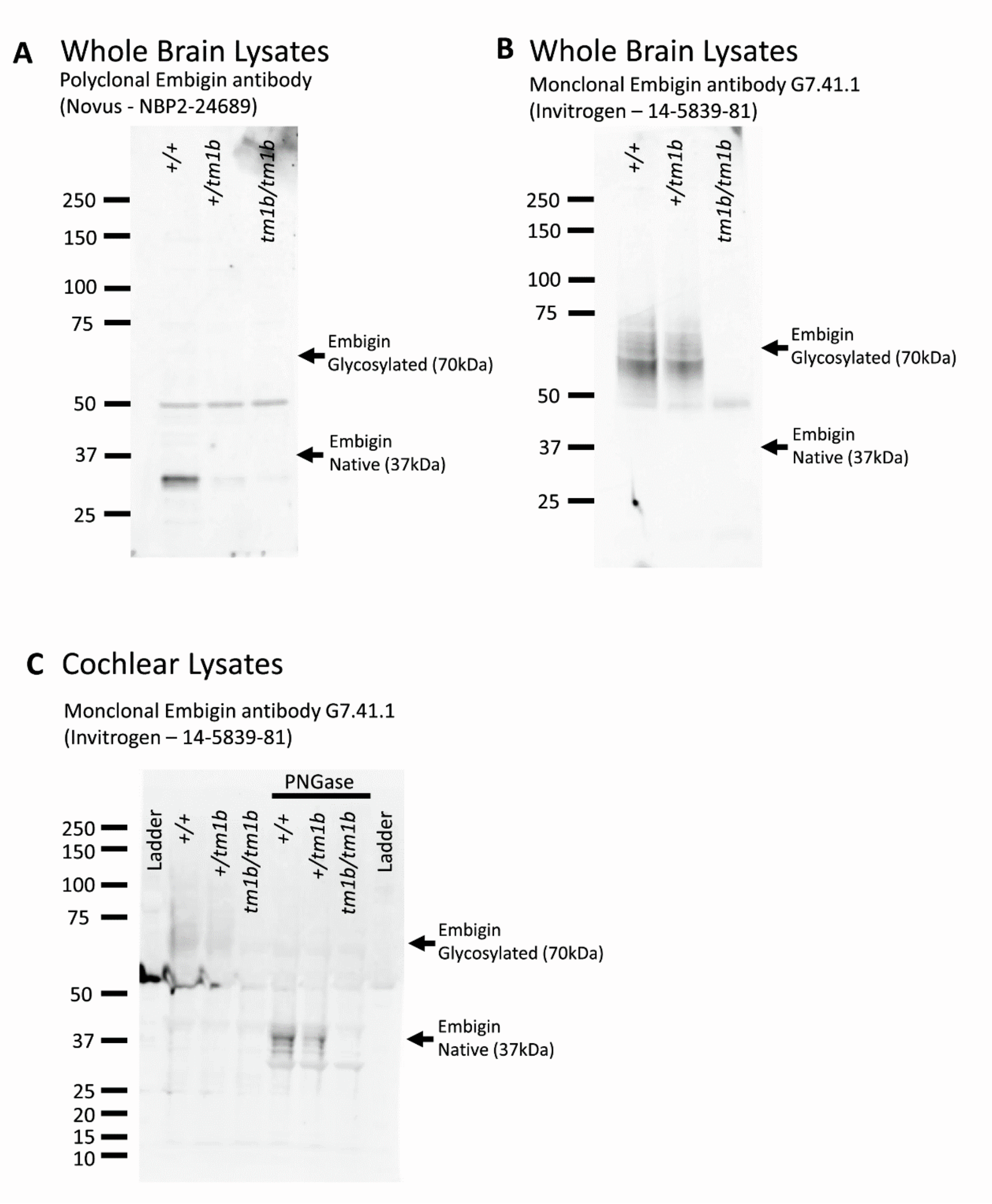

Fig S1. Validation of an Embigin antibody for Western Blot.
Related Figure 1. (A-B) $Emb^{+/+}$, $Emb^{+/tm1b}$, and $Emb^{tm1b/tm1b}$ whole brain lysates were prepared for the purpose of validating the (A) Novus (NBP2-24689) and (B) Invitrogen G7.41.1 (14-5839-81) anti-Embigin antibodies for western blot. The Novus anti-Embigin antibody (A) produced an IgG band at 50kDa, and a ~30kDa band which was more prominant in the $Emb^{+/+}$ sample. This band did not match the expected molecular weight for glycosylated (70kDa) or deglycosylated (native, 37kDa) Embigin, thus we considered this antibody to be non-specific. The monoclonal G7.41.1 anti-Embigin antibody (B) produced a diffuse signal in $Emb^{+/+}$ and $Emb^{+/tm1b}$ lysates, which was absent in the $Emb^{tm1b/tm1b}$ lysates, which was centred around the expected molecular weight for glycosylated Embigin (70kDa). (C) Cochlear $Emb^{+/+}$, $Emb^{+/tm1b}$, and $Emb^{tm1b/tm1b}$ lysates probed with the G7.41.1 antibody showed a weak, diffuse band at 70kDa in $Emb^{+/+}$, $Emb^{+/tm1b}$ lysates, which was shifted to ~37kDa when deglycosylated using PNGase F.

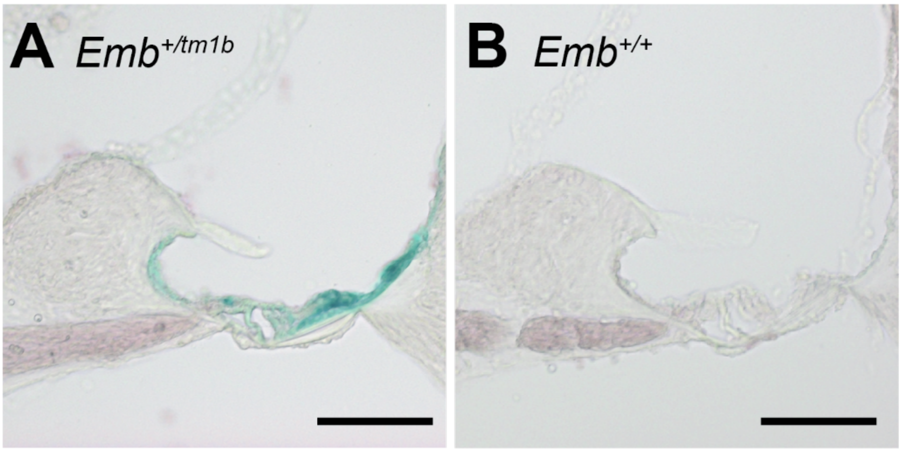

Fig S2. β-Gal staining is concordant with anti-Embigin immunolabelling.
Related to Figure 1. Cochlear cryosections prepared from 4-week old *Emb*$^{+/tm1b}$ (A) and *Emb*$^{+/+}$ (B) littermate mice stained with X-Gal (blue) and counterstained with nuclear fast red (pink). (A) In sections from *Emb*$^{+/tm1b}$ mice, β-Gal activity (blue) is detected within the same regions as labelled using the anti-Embigin antibody. (B) No β-Gal activity is detected in sections from *Emb*$^{+/+}$ mice (B). Scale bar = 50μm.

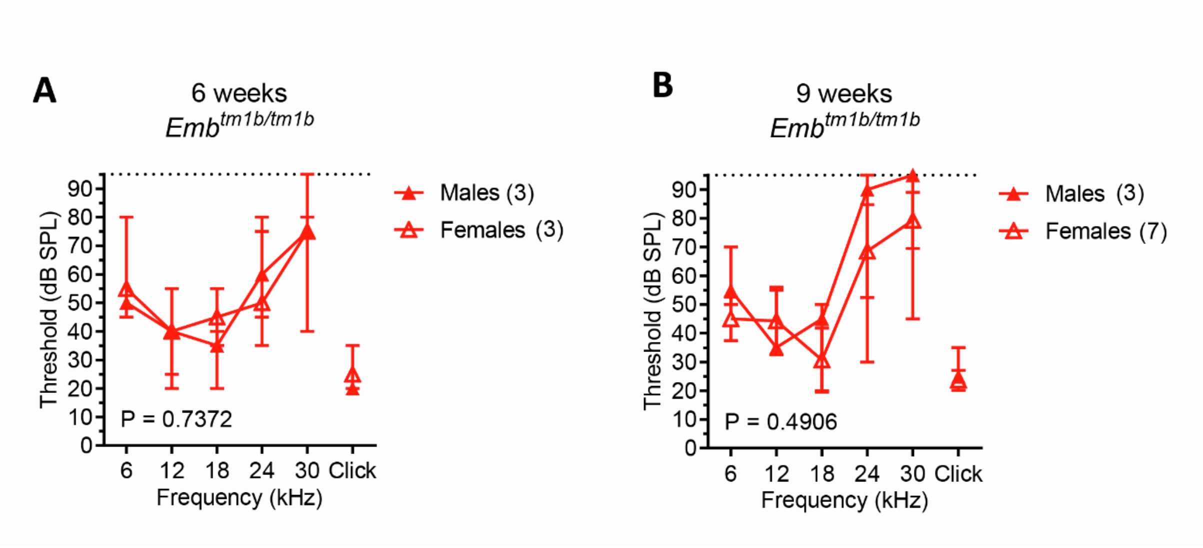

Fig S3. There is no difference in ABR threshold between male and female *Emb*$^{tm1b/tm1b}$ mice.
Related to Figure 2. ABR threshold measures recorded from male (solid triangle) and female (open triangle) *Emb*$^{tm1b/tm1b}$ (A) 6-week and (B) 9-week old mice maintained on a C57BL/6N genetic background. Comparison between male and female ABRs at these two timepoints showed no difference in the thresholds recorded. Data shown are median ± I.Q.R. Statistical analysis performed using a two-way ANOVA comparing against wild type with Holm-Šídák's test for multiple comparisons. Number of mice tested for each time point shown in brackets.

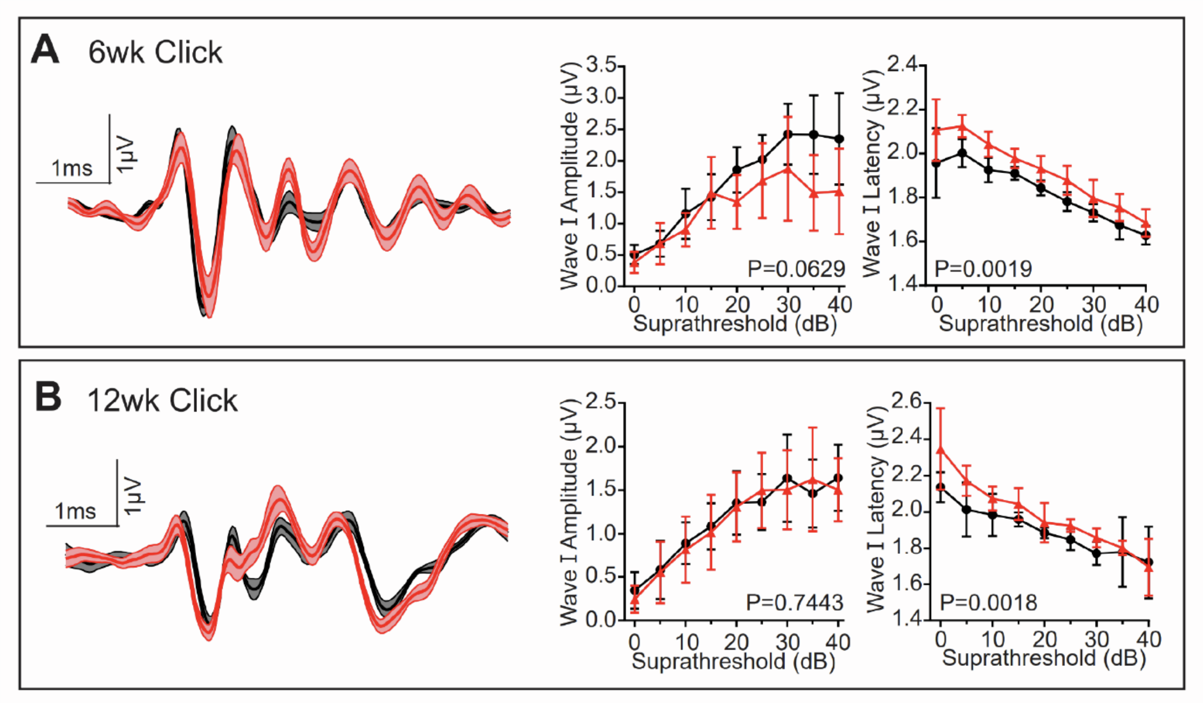

Fig S4. Suprathreshold ABR amplitudes are not altered in the *Emb$^{tm1b/tm1b}$* mutant.
Related to Figure 2. (A) Click-evoked ABR Wave I measures comparing *Emb$^{tm1b/tm1b}$* (red triangles, n = 6) with *Emb$^{+/+}$* (black circles, n = 8) mice at 6-weeks of age. Amplitudes were not significantly different (P = 0.0629). However, latencies are slightly delayed, which is more apparent at low intensities (P = 0.0019). (B) Click-evoked ABR Wave I measures comparing *Emb$^{tm1b/tm1b}$* (red triangles, n = 8) with *Emb$^{+/+}$* (black circles, n = 10) mice at 12- weeks of age. As with 6-weeks, amplitudes were not significantly different (P = 0.7443), however latencies are slightly delayed at low intensities (P = 0.0018). Statistical analysis performed using a two-way ANOVA comparing against wild type with Holm-Šídák's test for multiple comparisons. Insets show an average of the whole waveform following click stimulus at 30 dB above threshold.

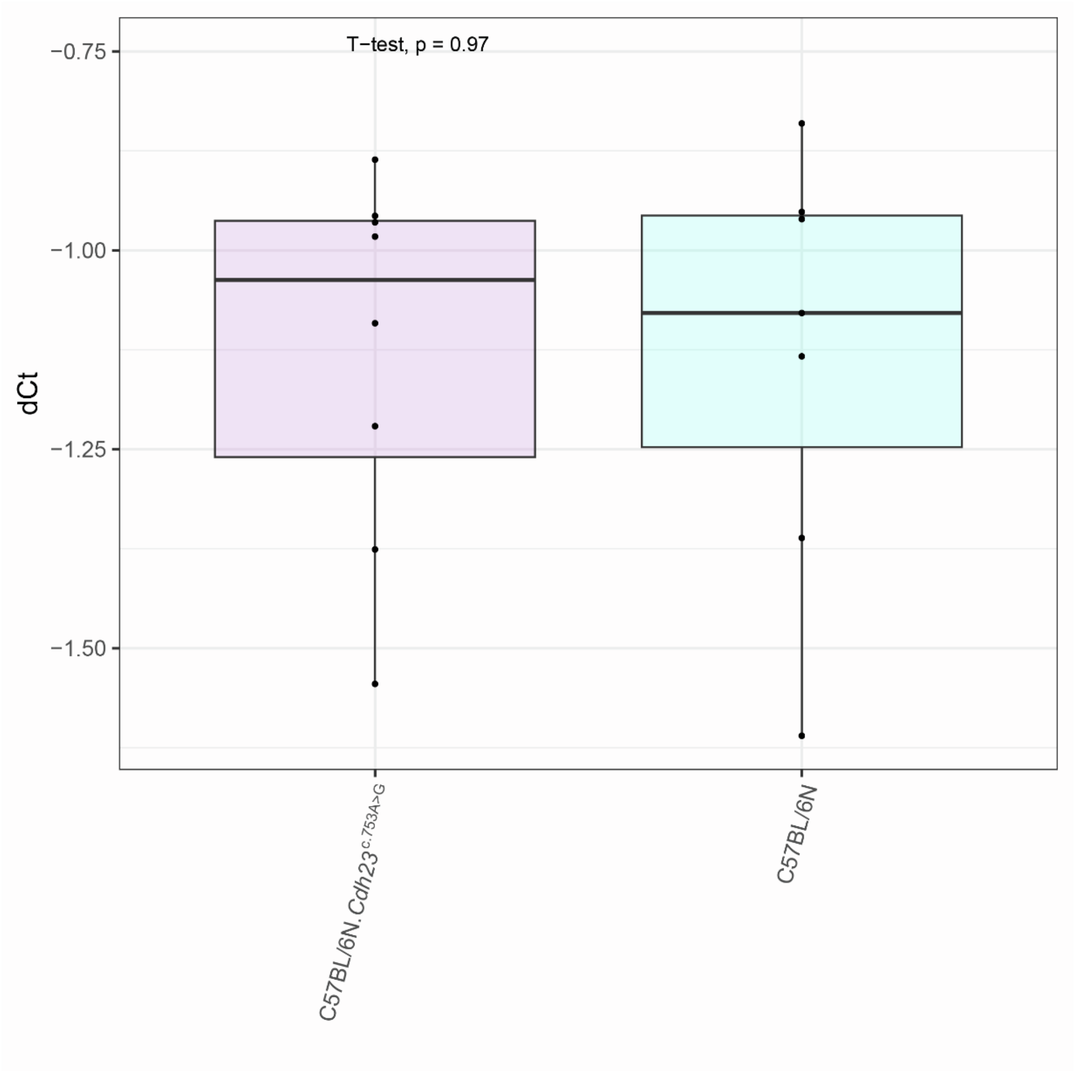

Figure S5. *Cdh23^{ahl}* allele does not affect *Embigin* expression in wild type mice. Related to Figures 2 and 3. Box-plot representing the expression of *Embigin* (*Emb*) in cochlear total RNA prepared from 7 'repaired' wild type (C57BL/6N.*Cdh23$^{c.753A>G}$*, pink) mice (3 males and 4 females) with 8 'standard' wild type (C57BL/6N, blue) mice (4 males and 4 females) at 4-weeks of age, evaluated by qPCR. Dots represent ΔCt values obtained from individual mice. Statistical analysis performed using a Student's un-paired T-test.
